# 4D flow cardiovascular magnetic resonance recovery profiles following pulmonary endarterectomy in chronic thromboembolic pulmonary hypertension

**DOI:** 10.1186/s12968-022-00893-x

**Published:** 2022-11-14

**Authors:** Melody L. Dong, Arshid Azarine, Francois Haddad, Myriam Amsallem, Young-Wouk Kim, Weiguang Yang, Elie Fadel, Laure Aubrege, Michael Loecher, Daniel Ennis, Jérôme Le Pavec, Irene Vignon-Clementel, Jeffrey A. Feinstein, Olaf Mercier, Alison L. Marsden

**Affiliations:** 1 Department of Bioengineering, Stanford University, Stanford, CA USA; 2 Department of Radiology, Groupe Hospitalier Paris Saint-Joseph, Paris, France; 3 Biomedical Engineering Lab, Groupe Hospitalier Paris Saint-Joseph, Paris, France; 4 Division of Cardiovascular Medicine, Stanford University, Stanford, CA USA; 5 Department of Pediatric Cardiology, Stanford University, Stanford, CA USA; 6 Department of Radiology, Stanford University, Stanford, CA USA; 7 Department of Thoracic Surgery, Marie Lannelongue Hospital, Université Paris-Saclay, Le Plessis Robinson, France; 8 Department of Respirology, Marie Lannelongue Hospital, Le Plessis Robinson, France; 9 Pulmonary Hypertension: Pathophysiology and Novel Therapies, Marie Lannelongue Hospital, INSERM UMR-S 999, Le Plessis Robinson, France; 10 Inria, Saclay Ile-de-France, Palaiseau, France; 11 Department of Bioengineering and Pediatric Cardiology, Stanford University, Stanford, CA USA

**Keywords:** Chronic thromboembolic pulmonary hypertension, Pulmonary endarterectomy, 4D flow magnetic resonance

## Abstract

**Background:**

Four-dimensional flow cardiovascular magnetic resonance imaging (4D flow CMR) allows comprehensive assessment of pulmonary artery (PA) flow dynamics. Few studies have characterized longitudinal changes in pulmonary flow dynamics and right ventricular (RV) recovery following a pulmonary endarterectomy (PEA) for patients with chronic thromboembolic pulmonary hypertension (CTEPH). This can provide novel insights of RV and PA dynamics during recovery. We investigated the longitudinal trajectory of 4D flow metrics following a PEA including velocity, vorticity, helicity, and PA vessel wall stiffness.

**Methods:**

Twenty patients with CTEPH underwent pre-PEA and > 6 months post-PEA CMR imaging including 4D flow CMR; right heart catheter measurements were performed in 18 of these patients. We developed a semi-automated pipeline to extract integrated 4D flow-derived main, left, and right PA (MPA, LPA, RPA) volumes, velocity flow profiles, and secondary flow profiles. We focused on secondary flow metrics of vorticity, volume fraction of positive helicity (clockwise rotation), and the helical flow index (HFI) that measures helicity intensity.

**Results:**

Mean PA pressures (mPAP), total pulmonary resistance (TPR), and normalized RV end-systolic volume (RVESV) decreased significantly post-PEA (P < 0.002). 4D flow-derived PA volumes decreased (P < 0.001) and stiffness, velocity, and vorticity increased (P < 0.01) post-PEA. Longitudinal improvements from pre- to post-PEA in mPAP were associated with longitudinal decreases in MPA area (r = 0.68, P = 0.002). Longitudinal improvements in TPR were associated with longitudinal increases in the maximum RPA HFI (r=-0.85, P < 0.001). Longitudinal improvements in RVESV were associated with longitudinal decreases in MPA fraction of positive helicity (r = 0.75, P = 0.003) and minimum MPA HFI (r=-0.72, P = 0.005).

**Conclusion:**

We developed a semi-automated pipeline for analyzing 4D flow metrics of vessel stiffness and flow profiles. PEA was associated with changes in 4D flow metrics of PA flow profiles and vessel stiffness. Longitudinal analysis revealed that PA helicity was associated with pulmonary remodeling and RV reverse remodeling following a PEA.

**Supplementary Information:**

The online version contains supplementary material available at 10.1186/s12968-022-00893-x.

## Background

Chronic thromboembolic pulmonary hypertension (CTEPH) is a rare and progressive form of pulmonary hypertension (PH) [1]. Persistent thrombi and obstructions of the proximal pulmonary arteries (PAs) in patients with CTEPH lead to abnormally elevated PA pressures, vascular remodeling, and eventually right heart failure if not treated [1]. For operable patients with CTEPH, a pulmonary endarterectomy (PEA), where obstructive thrombi are removed from the PAs, is the standard of care at experienced centers [2]. PEAs improve the outcomes in most patients with CTEPH, returning PA pressures and right ventricular (RV) function to near-normal values [2–7]. While cardiovascular magnetic resonance (CMR) provides characterization of RV reverse remodeling through volumetric parameters, quantification of blood flow dynamics in the PAs just distal to the RV can provide a significant understanding of the RV-PA dynamics that may inform new phenotypes of RV recovery in patients with CTEPH following a PEA.

Though current standards for evaluating the PAs in patients with CTEPH involve invasive procedures such as a right heart catheter to measure PA pressures, non-invasive 4D flow CMR (4D flow CMR) can provide time-resolved velocity and flow information in the PAs and the RV [8]. Previous studies have shown correlations between non-invasive 4D flow CMR-derived velocity profiles and invasively measured PA pressures, as well as changes in the peak velocities after surgical correction in patients with CTEPH [4, 6, 9–11]. Complex secondary flow structures such as vortical flow, where fluid rotates around an axis, and helical flow, where fluid rotates and moves in one direction in a cork-screw motion with clockwise (positive) or counterclockwise (negative) rotation, have been characterized with 4D flow CMRs of major vessels in healthy and diseased PAs and aortas, such as PH and bicuspid aortic valve disease [12–14]. Vorticity and recirculation of flow have been associated with inefficient blood flow and diseased states in the PAs and aorta [15, 16]. In Schafer et al’s study, helical flow quantified as the summation of both clockwise and counterclockwise helicity was lower in patients with PH [14]. However, this method of quantifying helicity may predominantly capture changes in velocity and can be the result of clockwise and counterclockwise helicity negating each other upon summation, especially in the presence of counter-rotating vortices known as Dean vortices, which tend to form in curved vessels [17]. Other methods focus on the balance between clockwise and counterclockwise helicity and normalized helicity intensity in aortic valvular disease [18, 19]. 4D flow CMR in the PAs has shown promise for characterizing RV-PA dynamics in healthy subjects and patients with PH through fluid dynamics derived metrics of secondary flow profiles, such as vorticity and helicity, specifically in severe stages of PH and large dilatation of the pulmonary trunk [12, 14, 20–23]. However, comprehensive analyses of velocity flow profiles and secondary flow profiles including vorticity and helicity have yet to be investigated in patients with CTEPH following a PEA to determine their utility in evaluating recovery and reverse remodeling of the RV.

The objective of our study was to investigate novel 4D flow CMR metrics of the PAs to characterize recovery and RV reverse remodeling following a PEA in patients with CTEPH. To facilitate and standardize 4D PA flow analytics, we developed a 4D flow CMR post-processing pipeline combining semi-automated segmentation of the PAs with integrated automatic extraction of 4D flow-derived PA volumes, velocity, and secondary flow metrics including novel measures of helicity and vorticity. We evaluated these 4D flow metrics in patients with CTEPH before and at least 6 months after a PEA to characterize recovery following a PEA. These metrics show the potential of 4D flow CMR to non-invasively capture RV reverse remodeling and longitudinal recovery in patients with CTEPH with hemodynamic changes occurring between the RV and the PAs.

## Methods

### Study cohort

Patients with CTEPH were enrolled in the PRINCEPT study as part of an approved IRB protocol (NCT03205085, IRB approval 2017-A00785-48) at the Marie Lannelongue Hospital where the PEAs were performed. A total of 73 patients were enrolled initially, of which, 20 patients had CMR including a 4D flow CMR immediately before the PEA and at least 6 months after the PEA (average 7.5 ± 1.3 months) with clear anatomic image resolution of the PAs which were used for volume and area metrics. From these patients, 15 had high quality velocity resolution for both pre-PEA and post-PEA scans that satisfied conservation of flow from the main PA (MPA) to the left and right PAs (LPA, RPA) with a < 20% error which were used for velocity-based metrics capturing velocity flow profiles and secondary flow profiles. Of the 20 patients with satisfactory CMR, 18 had pre- and post-PEA measurements of mean PA pressure (mPAP) and total pulmonary resistance (TPR) by right heart catheter (RHC) procedures performed 68 ± 93 and 22 ± 46 days before the CMR scan at pre- and post-PEA timepoints. A group of 8 patients with mild to moderate valvular disease, but with no evidence of PH, RV systolic dysfunction (RV ejection fraction, RVEF > 50%), left ventricular (LV) systolic dysfunction (LV ejection fraction, LVEF > 50%) were used from the SUBCLAR study (NCT03549091) for comparison. Details and results of this control group are included in **Additional Files 13–17** for reference. Additional patient details can be found in Table 1.

### CMR acquisition

For all patients with CTEPH, CMR scans were performed on a 1.5 T CMR system (Optima MR 450 W, General Electric Healthcare, Waukesha, Wisconsin, USA) with a 36- or 48-channel coil for cardiac imaging. Short axis and long axis balanced steady-state free precession (bSSFP) cine images were acquired to assess RV volumes and function. Respiratory and electrocardiogram (ECG) gated 4D flow CMR sequences were acquired in a coronal 3D volume with full coverage of the thorax with an average acquisition time of 8.33 min (range 6.5–12 min). Velocity encoding (Venc) was set at 160–300 cm/s and 20–30 cardiac frames were acquired per cardiac cycle with a spatial resolution of 1.4–1.8 × 1.4–1.8 × 1.1–1.4 mm^3^.

From CMR short axis cine bSSFP images, RVEF was calculated from segmentation of RV end-systolic and end-diastolic volumes (RVESV, RVEDV, respectively) excluding trabeculations to account for RV muscularization (Arterys Inc., San Francisco, California, USA).

**Table 1:** Patient characteristics and outcomes

	CTEPH (n = 20)
Age (years)	62 +/- 14
Male	12 (60%)
Height (cm)	171 +/- 10
Weight (kg)	76 +/- 16
BSA (m^2^)	1.9 +/- 0.2
BMI	26.1 +/- 4.0
Time between PEA and post-PEA 4D flow CMR (months)	7.5 +/- 1.3
San Diego Classification	I = 2 (10%) II = 13 (65%) III = 4 (20%) Not recorded = 1 (5%)
Obesity (BMI > 30 kg/m^2^)	3 (15%)
Diabetes mellitus	2 (10%)
Coronary Artery Disease	1 (5%)
Hypertension	14 (70%)

Values are mean ± standard deviation or n (%).* BSA*, body surface area; *BMI*, body mass index

### Semi-automated 4D flow CMR post-processing pipeline

**Fig. 1:**
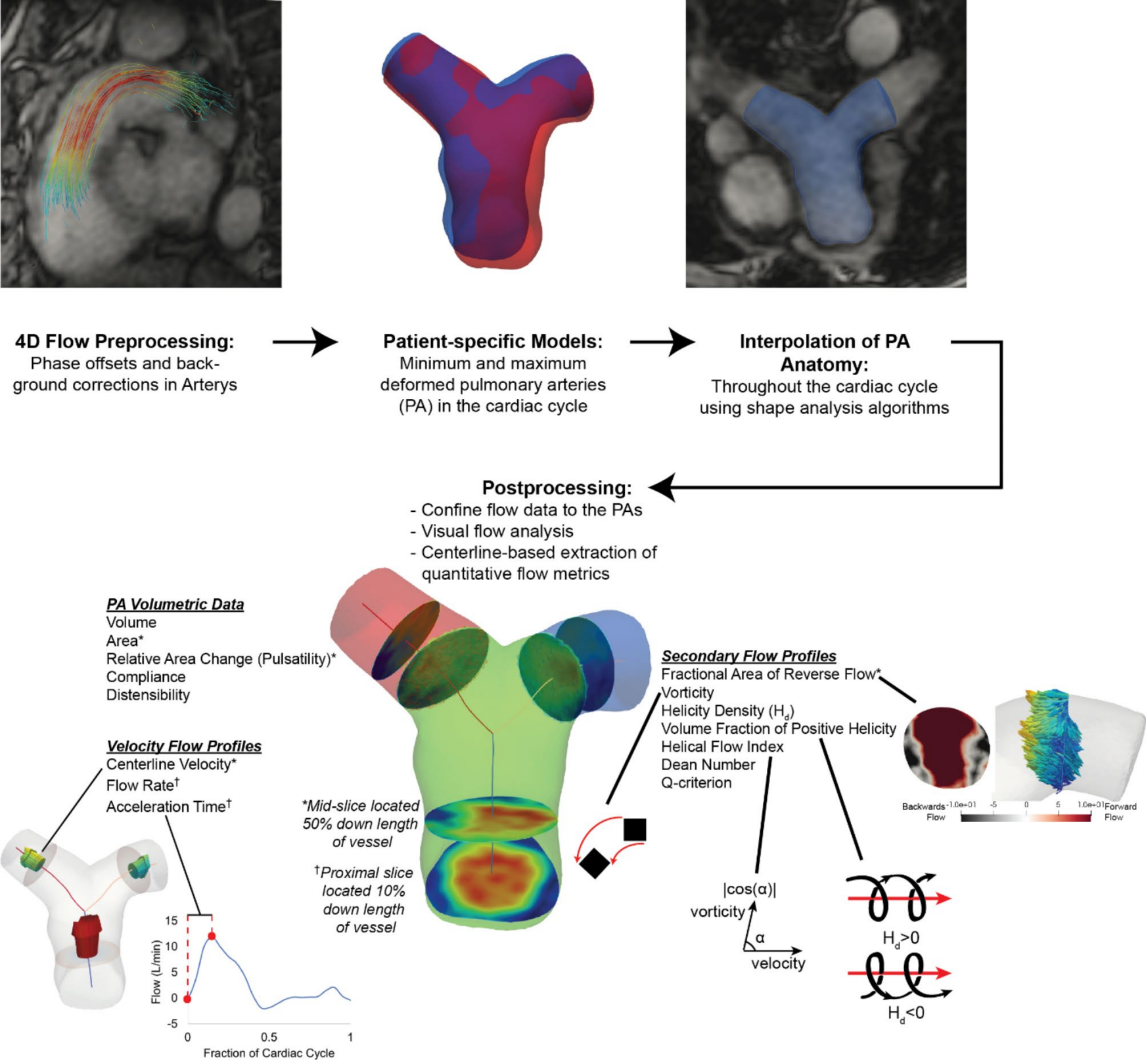
Semi-automated pipeline to extract velocity from 4D flow cardiovascular magnetic resonance (4D flow CMR) contained solely within the pulmonary arteries through image segmentation and shape analysis algorithms as well as automated computation of velocity metrics from branches and cross-sectional slices throughout the cardiac cycle. Visualization of the 4D flow CMR-derived metrics extracted from the pulmonary arteries (PAs) show illustrative explanations of acceleration time ratio, fractional area of reverse flow, vorticity, helicity, and helical flow index. * = metric was computed from a mid-slice located 50% down the length of the vessel. ^†^ = metric was computed from a proximal slice located 10% down the length of the vessel. All other metrics were computed from the volumes of the main, left, and right PA branches

To analyze the velocity and anatomic data of the PAs from the 4D flow CMR, we developed a semi-automated 4D flow CMR post-processing pipeline using custom Python scripts (Fig. 1). All 4D flow CMR data were uploaded and stored using a medical imaging cloud platform, Arterys Cardio AI (Arterys Inc.), where phase offset and background corrections were applied. The corrected velocity and 4D CMR images were downloaded and further post-processed for image and velocity analysis using custom Python scripts. To confine the 4D flow velocity to the PAs, we created 3D models of the proximal lobar PAs using the open-source software, SimVascular (simvascular.org) [24]. Models extended from the pulmonary valve to the LPA and RPA and were truncated at the first segmental branching point on both the left and right PAs from the 4D CMR magnitude images. Because the PAs translate, distend, and expand throughout the cardiac cycle, we created 3D models at the minimum and the maximum deformed timepoints of the PAs. The shape analysis registration algorithm from Deformetrica (deformetrica.org) [25] was then used to create interpolated geometries for all timepoints in the cardiac cycle between the minimum and maximum deformed timepoints using a kernel width of 1. The interpolated geometries were visually checked with the 4D flow images to validate accurate interpolation of the PA anatomy throughout the entire cardiac cycle. If the interpolated PA geometries did not align with the 4D flow image, additional 3D models were created, and new PA geometries were interpolated until the PA geometries matched the image for all timepoints. All geometries were discretized into 1.2 mm tetrahedral elements to match the minimum resolution of the 4D flow images. Velocities from the 4D flow CMR dataset were resampled onto the mesh with a linear interpolation. To automate extraction of hemodynamic metrics from the PAs, centerlines were generated for each PA geometry at each timepoint using the Vascular Modeling ToolKit (vmtk.org) [26]. The MPA, LPA, and RPA branch regions were identified from the centerlines, and two cross-sectional slices perpendicular to the centerlines were taken 10% and 50% down the length of each branch to capture the flows coming directly into each branch and to compute hemodynamics in the middle of each branch. Branch regions were defined as the volume region between the caps of the PA geometry to the bifurcation region where the centerlines from the LPA and RPA intersected the MPA.

### Hemodynamic metrics computed

Within the semi-automated post-processing pipeline, we computed metrics describing volumetric data, velocity flow profiles, and secondary flow profiles of the PAs. Details of the metrics computed are shown in Fig. 1 with illustrations of some 4D flow CMR-derived PA metrics. A table of all metrics computed is listed and derived in Table 2. Maximums, minimums, and time averages were computed for each metric throughout the cardiac cycle and during the systolic and diastolic periods, defined by the end-diastolic and end-systolic timepoints. Percent predicted RV metrics were computed to adjust for sex, age, weight, and height (Table 2) [27]. Volumetric data including MPA, LPA, and RPA vessel volumes and mid-vessel cross-sectional areas as well as relative area change (RAC), compliance, and distensibility capturing PA wall stiffness were computed.

To capture velocity flow profiles, centerline velocities, flow rates, and acceleration time ratio were computed for the MPA, LPA, and RPA (Table 2). Centerline velocities were computed as the largest velocity within the center area of a cross-sectional mid-slice located 50% down the length of each branch. The center area of the slice was defined by a radius that was 30% of the total cross-sectional radius of the branch. Flow rates and waveforms were computed from a cross-sectional proximal slice located 10% down the length of each branch to account for entrance effects. The acceleration time ratio was computed from these flow waveforms. For all 4D flow CMR scans, conservation of flow was computed as the error between the flow in the MPA to the sum of the flows in the LPA and RPA.

To capture secondary flow structures such as recirculation, flow separation, and swirling flow in the PAs, we quantified areas of reverse flow, vorticity, and helicity. To capture recirculation, the fractional area containing reverse flow, defined by any velocity vector that is perpendicular or negative to the forward flow direction, was computed for a cross-sectional slice located in the middle of each branch.

Vorticity is defined as the curl of the velocity quantifying the rotation of individual fluid particles as it moves with velocity and may be found in visible vortex structures, swirling flow, and shearing against the vessel walls and within blood flow [28] (Table 2). Vorticity in the MPA, LPA, and RPA branch volumes were computed as spatially and temporally averaged values over the cardiac cycle and systolic period.

Helicity density (${H}_{d}$) quantifies the helical flow or flow with a corkscrew-like motion in one direction and is proportional to the velocity and the vorticity (Table 2). The sign of H_*d*_ describes if flow rotating around an axis in one direction has a clockwise (positive helicity, ${H}_{d}$>0) or counterclockwise (negative helicity, ${H}_{d}$<0) rotation where vorticity is pointing in the same or opposite direction of the velocity, respectively. This is relevant when flow is moving in one direction and does not distinguish between flow moving in a forward or reverse direction. In a curved vessel, secondary flow structures with counter-rotating vortices known as Dean vortices can develop, containing a balance of positive and negative helicity. The propensity to develop these secondary flow structures is dependent on Dean number (De), a dimensionless parameter quantifying the inertial, centripetal, and viscous forces resulting in a curved tube. To understand the balance between positive and negative helicity in the PAs, we computed the volume fraction of the MPA, LPA, and RPA that contained positive helicity (${H}_{d}$>0). Because large differences in velocity between patients affect ${H}_{d}$, the helical flow index (HFI), was used to normalize the helicity to the velocity and vorticity and is defined as the absolute value of the cosine of the angle between the vorticity and velocity, limiting the range from 0 to 1, and capturing the relative intensity of helicity [18, 19].

**Table 2:** Summary of 4D flow-derived metrics

Category	Metric	Definition	Comment
Geometry & Stiffness	PA volume, *V* (ml)	Temporally averaged volume of the MPA, LPA, RPA branches over a cardiac cycle	Internal diameters potentially influenced by proximal thrombus
	PA area, *A* (cm^2^)	Centerline-based cross-sectional slice located 50% down the length of the MPA, LPA, and RPA branches	
	Relative area change (Pulsatility)	$\frac{{A}_{\text{m}\text{a}\text{x}}-{A}_{\text{m}\text{i}\text{n}}}{{A}_{\text{m}\text{i}\text{n}}}$	
	Compliance (cm^2^/mmHg)	$\frac{{A}_{\text{m}\text{a}\text{x}}-{A}_{\text{m}\text{i}\text{n}}}{SPAP-DPAP}$	Healthy PA compliance = 0.15 cm^2^/mmHg^§^
	Distensibility (%/mmHg)	$\frac{Relative Area Change}{SPAP-DPAP}\times 100$	Healthy PA distensibility = 3.1%/mmHg^§^
Velocity Flow Profile	Centerline velocity (cm/s)	Maximum velocity in the center 30% radius of a cross-sectional slice located 50% down the length of each branch.	
	Flow rate (L/min)	$\int \left(\overrightarrow{u}\cdot \overrightarrow{n}\right)dA$ $\overrightarrow{u}$ is the velocity vector, $\overrightarrow{n}$ is the surface normal vector, A is the area of the cross-sectional slice located 10% down the length of the MPA, LPA, and RPA	
	Acceleration ime ratio	$$\frac{{t}_{end-diastole}-{t}_{peak systolic flow}}{{t}_{systole}}$$ $${t}_{systole}$$ is the time between the end-systolic and end-diastolic timepoint	
Secondary Flow Profile	Fractional area of reverse flow	Areas where velocity is perpendicular or negative to the forward flow direction. $\frac{{{A_{\vec u \cdot \vec n \leqslant 0}}}}{{{A_{total}}}}$ Taken at a cross-sectional slice 50% down the length of the branch	Quantifies areas of recirculation, may correspond to large visual vortices
	Vorticity, $\overrightarrow{\omega }$ (1/s)	$\frac{1}{\text{V}}\int \nabla \times \overrightarrow{u} dV$ $\nabla$ is the spatial gradient, $\overrightarrow{u}$ is the velocity vector, V is the volume of the branch; spatially averaged over the volume of the branch	Local rotation in a fluid flow including local shearing in space
	Helicity density, *H*_*d*_ (m/s^2^)	${H_d} = \vec u \cdot \vec \omega$ Amount of rotation parallel to the local velocity vector	Clockwise rotation when ${H}_{d}$>0, counterclockwise rotation when ${H}_{d}$<0
	Volume fraction of positive helicity	$\frac{{V}_{{H}_{d}>0}}{{V}_{total}}$ V is the volume of the branch	
	Helical flow index, HFI	$\frac{1}{V}\int {\left( {\left| {\frac{{\vec u \cdot \vec \omega }}{{\left| {\vec u} \right|\left| {\vec \omega } \right|}}} \right|} \right)} dV$ Absolute value of the cosine of the angle between the vorticity and velocity vector, measure of helicity intensity	Limit from 0 to 1
	Dean number, *De*	$\frac{\rho \stackrel{-}{u}D}{\mu }\sqrt{\frac{D}{2{R}_{c}}}$ D is the diameter of the vessel, $\stackrel{-}{u}$ is the mean velocity (computed at a cross-sectional slice 50% down the length of the pulmonary trunk), $\rho$ is the density of blood (1.06 g/cm^3^), $\mu$ is the viscosity of blood (0.04 Poise), and ${R}_{c}$ is the radius of curvature of the vessel defined from the centerlines in the MPA, LPA, or RPA	Non-dimensional parameter of the inertial, centripetal, and viscous forces; tendency of Dean vortices to form in a curved vessel
	Q-criterion	$\frac{1}{2}\left[ {\parallel \Omega {\parallel ^2} - \parallel S{\parallel ^2}} \right] > 0$ Defines a vortex as a connected fluid region where the norm of the vorticity tensor, ${\Omega }$, outweighs the norm of the strain rate tensor, $S$.	May not uniquely detect visual vortical regions
RV Function^§§^	% Predicted RV metrics	$\frac{RV metric}{m\cdot Ag{e}^{a}\cdot H{t}^{b}\cdot W{t}^{c}}$	Adjusted for differences in sex, age, weight, height
		RVEF: *a* = 0.0706, *b*=-0.00771, *c*=-0.0782 * m* = 75.19 (female), 71.52 (male)	
		RVEDV: *a*=-0.258, *b* = 1.582, *c* = 0.382 * m* = 27.94 (female), 31.50 (male)	
		RVESV: *a*=-0.417, *b* = 1.501, *c* = 0.617 * m* = 5.58 (female), 7.24 (male)	
		RVSV: *a*=-0.187, *b* = 1.574, *c* = 0.304 * m* = 21.12 (female), 22.42 (male)	

^§^Healthy PA compliance and distensibility from Sanz et al. [29]

^§§^RV metrics were adjusted for sex, age, weight, and height based on normalized equations from Kawut et al. [27]. *RV*, right ventricle, *EF*, ejection fraction, *EDV*, end-diastolic volume, *ESV*, end-systolic volume, *SV*, stroke volume, *PA*, pulmonary artery

### Simulations of volume changes

To further explain the physical changes in flow from pre-PEA to post-PEA, computational fluid dynamics simulations were used to control for changes in flow and volume that occur from pre-PEA to post-PEA timepoints (**Additional file 1**). A representative PA geometry, flow waveform, and approximate PA pressures from one of the post-PEA patients with CTEPH (PH2) was used to define a patient-specific PA anatomy and boundary conditions. Blood flow was simulated by solving the 3D Navier-Stokes equations using the open-source solver in SimVascular (simvascular.org) [24]. The post-PEA flow waveform was imposed at the MPA inlet and 3-element Windkessel boundary conditions, representing downstream resistance and compliance, were imposed at the outlets of the LPA and RPA and tuned to achieve approximate mean, systolic, and diastolic PA pressures of 18, 25, and 15 mmHg at the MPA, respectively. To capture the changes in the PA walls over the cardiac cycle, a deformable wall model was imposed with an elastic modulus of 250 kPa using the coupled momentum method [30] to represent the wall as a linear elastic membrane to achieve the patient-specific relative area change of 0.05 for patient PH2. To understand the hemodynamic changes involved in a larger volume, similar to a pre-PEA condition, we increased the PA geometry by 25% of the original volume with the same flow imposed to mimic the approximate average change in volume from pre- to post-PEA; similarly, a 25% increase in flow with the original PA geometry was used to understand changes from larger flows that might occur with patient variation. All hemodynamic metrics mentioned previously were extracted from the simulated flows for a volume 25% larger than the original post-PEA volume (representative of a pre-PEA condition), the original post-PEA volume and flow (representative of a post-PEA condition), and a flow 25% larger than the original post-PEA flow (representative of an alternative variation in a post-PEA condition).

### Statistical analysis

To determine significant differences in continuous variables between patients with CTEPH pre-PEA and post-PEA, a paired t-test was used with a p-value of 0.05 for significance. A Shapiro-Wilk test was used to test for normal distribution. Welch’s t-test was used to determine significant differences in continuous variables between patients with CTEPH pre-PEA and post-PEA. A Wilcoxon signed-rank test was used to determine significance in the New York Heart Association (NYHA) class between the pre-PEA and post-PEA timepoints. For correlations of 4D flow CMR-derived metrics with hemodynamic and RV remodeling metrics, Spearman’s correlation coefficient (r) was computed at baseline for all pre-PEA and post-PEA points. To determine longitudinal changes between 4D flow CMR-derived metrics and mPAP, TPR, and RV remodeling metrics, Spearman’s correlation coefficient was computed for changes in metrics from pre-PEA to post-PEA (i.e. *X*_post−PEA_ –*X*_pre−PEA_). All statistical analysis was performed using the open-source R software package [31].

## Results

### Endarterectomy specimens

During the PEA procedure, specimens of the clots removed from the PAs from patients with CTEPH were classified into three types based on the University of California, San Diego classification scale [32]: Type I - major vessel obstruction at the opening of the PAs, Type II - no major vessel thrombus, only thickened intima, and webs in the main, lobar, or segmental vessels, and Type III - distal PA obstruction in segmental and subsegmental branches with no occlusions. **Additional file 2** shows the images of these specimens immediately after removal for 15 out of 20 patients with CTEPH in the cohort and represent the approximate distribution of types for all patients with CTEPH.

### PA hemodynamic and RV remodeling changes

**Fig. 2:**
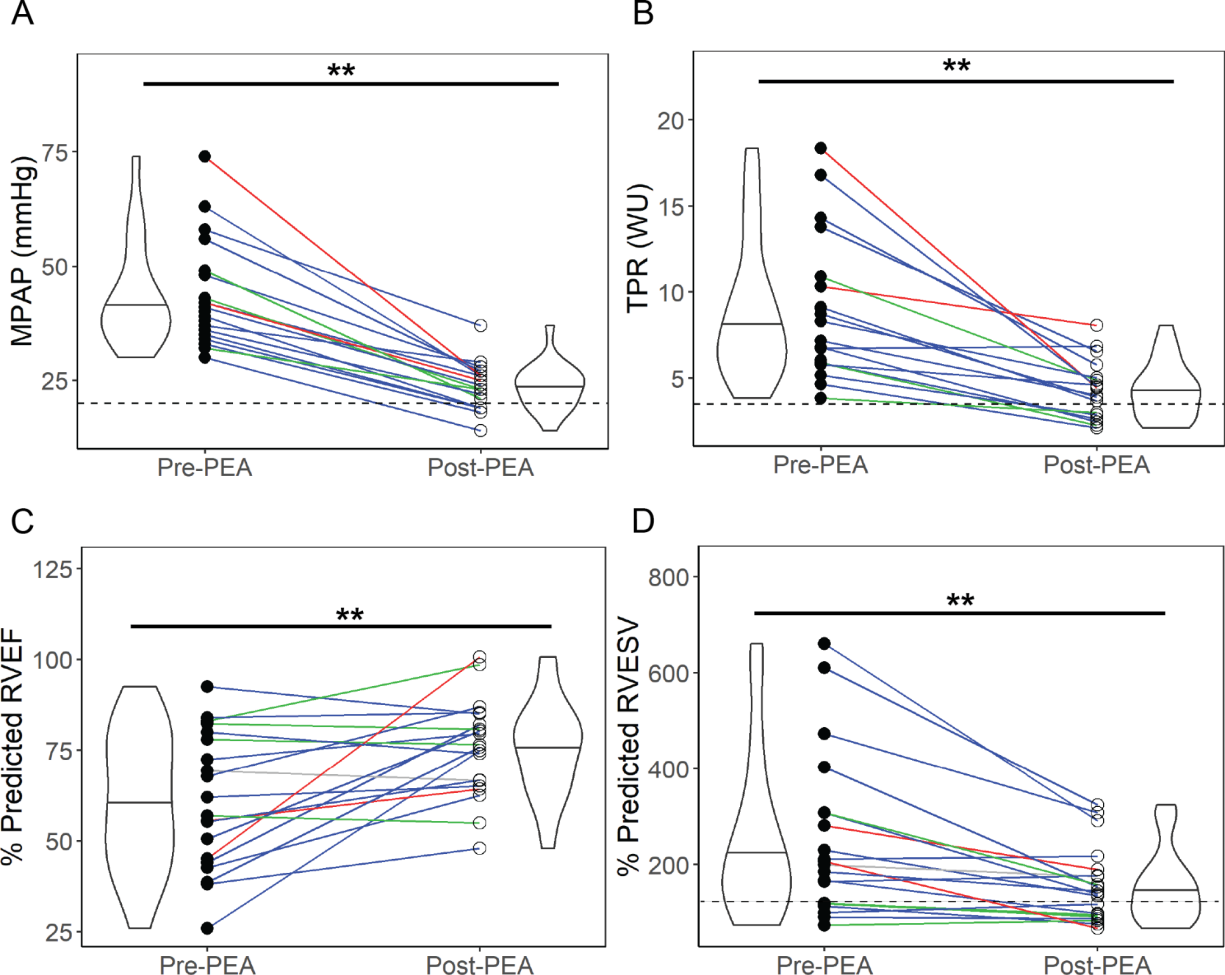
Hemodynamic and RV outcomes recovered to normal values in most patients with CTEPH post-pulmonary endarterectomy (PEA) for (a) mean pulmonary artery pressure (mPAP), (b) total pulmonary resistance (TPR), (c) % predicted right ventricular ejection fraction (RVEF), and (d) % predicted right ventricular end-systolic volume (RVESV) adjusted by sex, age, weight, and height [27]. Colored lines represent the San Diego classification of the PEA specimens for each patient – red = Type I, blue = Type II, green = Type III, grey = not recorded. Dotted lines in (a), (b), and (d) indicate healthy values of mPAP (< 20 mmHg), TPR (~ 3.5 WU), and % predicted RVESV (100%). Significant differences by t-test represented by *=p < 0.05, **=p < 0.01

PA hemodynamic metrics and RV metrics recovered significantly post-PEA towards accepted healthy values (Fig. 2) with all details listed in **Additional file 3**. Specifically, mPAP and TPR reduced significantly post-PEA with 28% of patients with CTEPH recovering healthy mPAP levels < 20 mmHg. RV metrics also improved significantly post-PEA in most patients with CTEPH with increases in % predicted RVEF and decreases in % predicted RVESV and RVEDV, showing RV reverse remodeling post-PEA. 79% of patients with CTEPH (15 out of 19) had an RVEF > 45% post-PEA. Functionally, patients with CTEPH showed significant improvement with a 15% increase in their 6-minute walk test distance and a decrease in NYHA functional class of heart failure. A quality check of the 4D flow CMR and CMR segmentations showed strong correlations between 4D flow MPA flow volumes and RV stroke volumes for all pre-PEA and post-PEA scans with a coefficient of determination of 0.94. A Bland-Altman plot showed fair agreements within 20 ml except for patients who presented with moderate/severe tricuspid regurgitation (**Additional file 4**).

### PA volume and wall stiffness changes

**Fig. 3:**
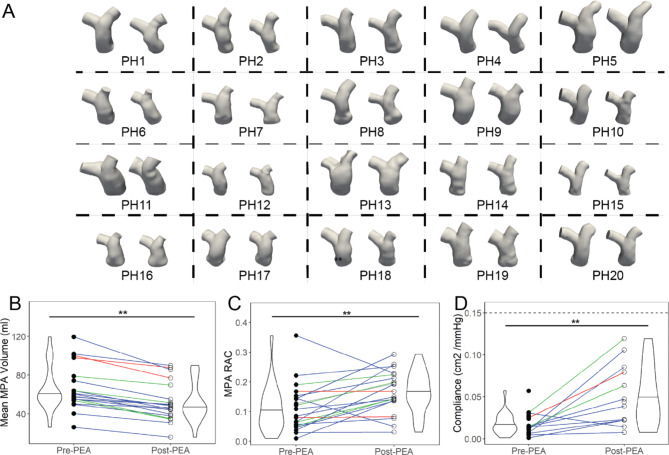
Volume and PA wall indices of patients with CTEPH changed post-PEA. (a) PA anatomies confined from the pulmonary valve to the first branch point in the left pulmonary artery (LPA) and right pulmonary artery (RPA) are shown at minimum volumes in the cardiac cycle for all patients with CTEPH (n = 20, first three rows, pre-PEA left, post-PEA right per box). Volumes of the (b) MPA decreased significantly post-PEA in patients with CTEPH. PA wall indices increased for the MPA (c) relative area change (RAC) and (d) compliance. The dotted line in (d) indicates healthy values of compliance (0.15 cm^2^/mmHg) [29]. Colored lines represent the San Diego classification of the PEA specimens for each patient – red = Type I, blue = Type II, green = Type III, grey = not recorded. *=p < 0.05, **=p < 0.01

PA volumes and wall stiffness indices changed significantly post-PEA (Fig. 3). A summary of the statistical analysis for all 4D flow CMR-derived metrics is listed in **Additional file 5**. Segmentation of the 3D PA geometries pre- and post-PEA were limited to the region from the pulmonary valve to the first bifurcation in the LPA and RPA branches (Fig. 3a). The volumes of the MPA (Fig. 3b), LPA, and RPA branches decreased significantly from pre-PEA to post-PEA. There was a slight increase in the LPA and RPA volumes for two patients with San Diego classification I (PH13 and PH9) due to clots in the proximal PAs not captured in the pre-PEA 4D flow CMR due to lack of blood flow and contrast in those regions. Corresponding to decreases in volume, the mean MPA, LPA, and RPA area all decreased post-PEA. Deformability and stiffness of the PA walls were characterized by the RAC, compliance (Fig. 3c, d), and distensibility of the MPA. RAC increased significantly post-PEA; compliance and distensibility increased significantly post-PEA towards values found in healthy patients.

### Velocity flow profile

**Fig. 4:**
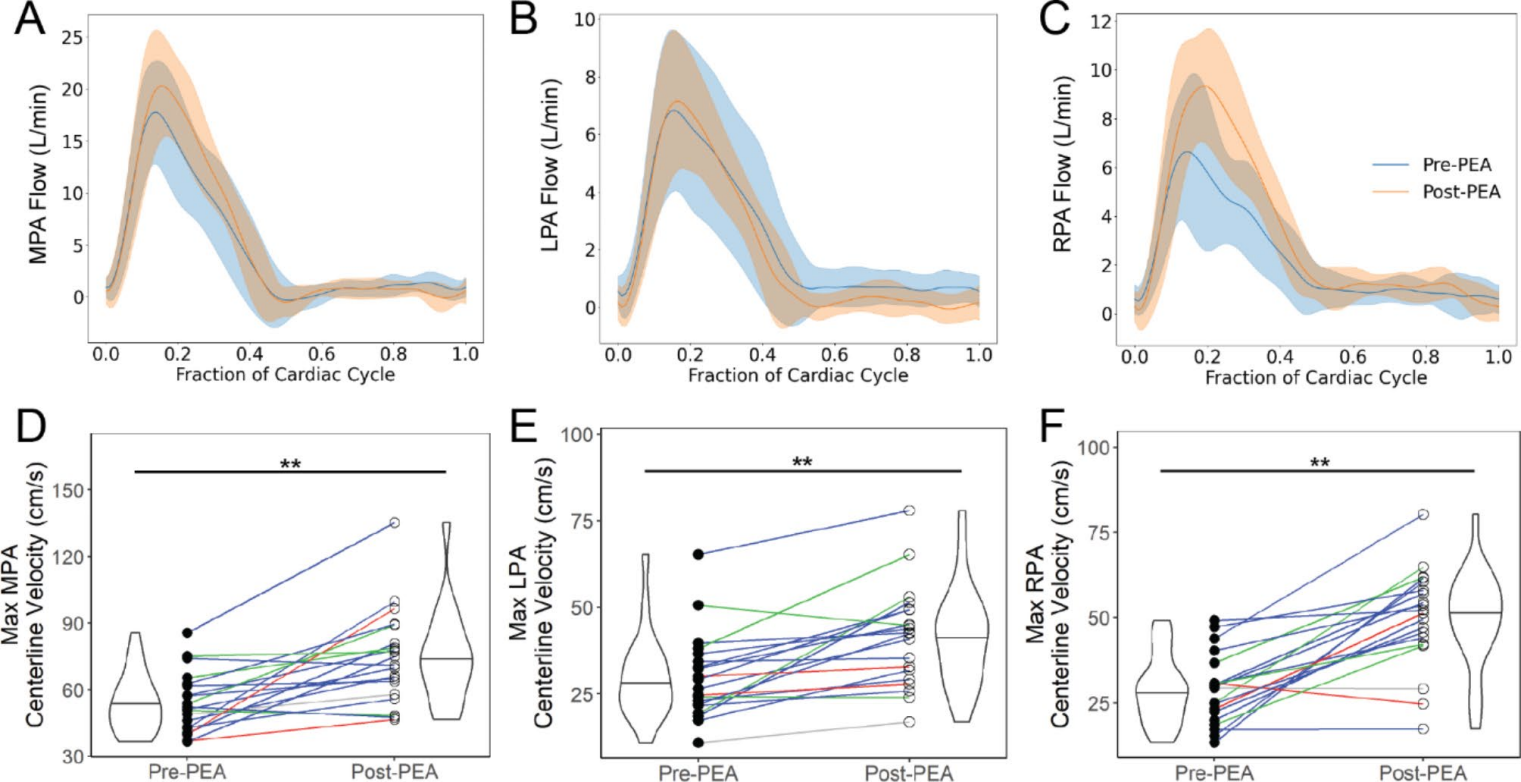
Flow waveforms averaged for all patients with CTEPH with high quality 4D flow CMR velocity (n = 15) pre-PEA (blue), post-PEA (orange) in the (a) MPA, (b) LPA, and (c) RPA where the solid line is the average, and the shaded region is the standard deviation. Centerline velocities in the (d) MPA, (e) LPA, and (f) RPA increased significantly post-PEA. Colored lines represent the San Diego classification of the PEA specimens for each patient – red = Type I, blue = Type II, green = Type III, grey = not recorded. Significant differences between groups denoted by: *=p < 0.05, **=p < 0.01

PA blood velocities increased significantly post-PEA (details in **Additional file 5**). In contrast, there was no significant increase in total flow rate in the MPA post-PEA as shown in the average patient flow waveforms pre- and post-PEA in Fig. 4a-c. Post PEA, there were no significant changes in the LPA, and slight increases in the RPA flow rate. With a large decrease in the volume, the centerline velocities in the MPA, LPA and RPA increased significantly from pre-PEA to post-PEA (Fig. 4d-f). The post-PEA centerline velocities were significantly greater than the pre-PEA timepoint. There was a significant increase in the RPA acceleration time ratio from pre-PEA to post-PEA. There was no change in the MPA and LPA acceleration time ratio from pre-PEA to post-PEA.

### Secondary flow profile

**Fig. 5:**
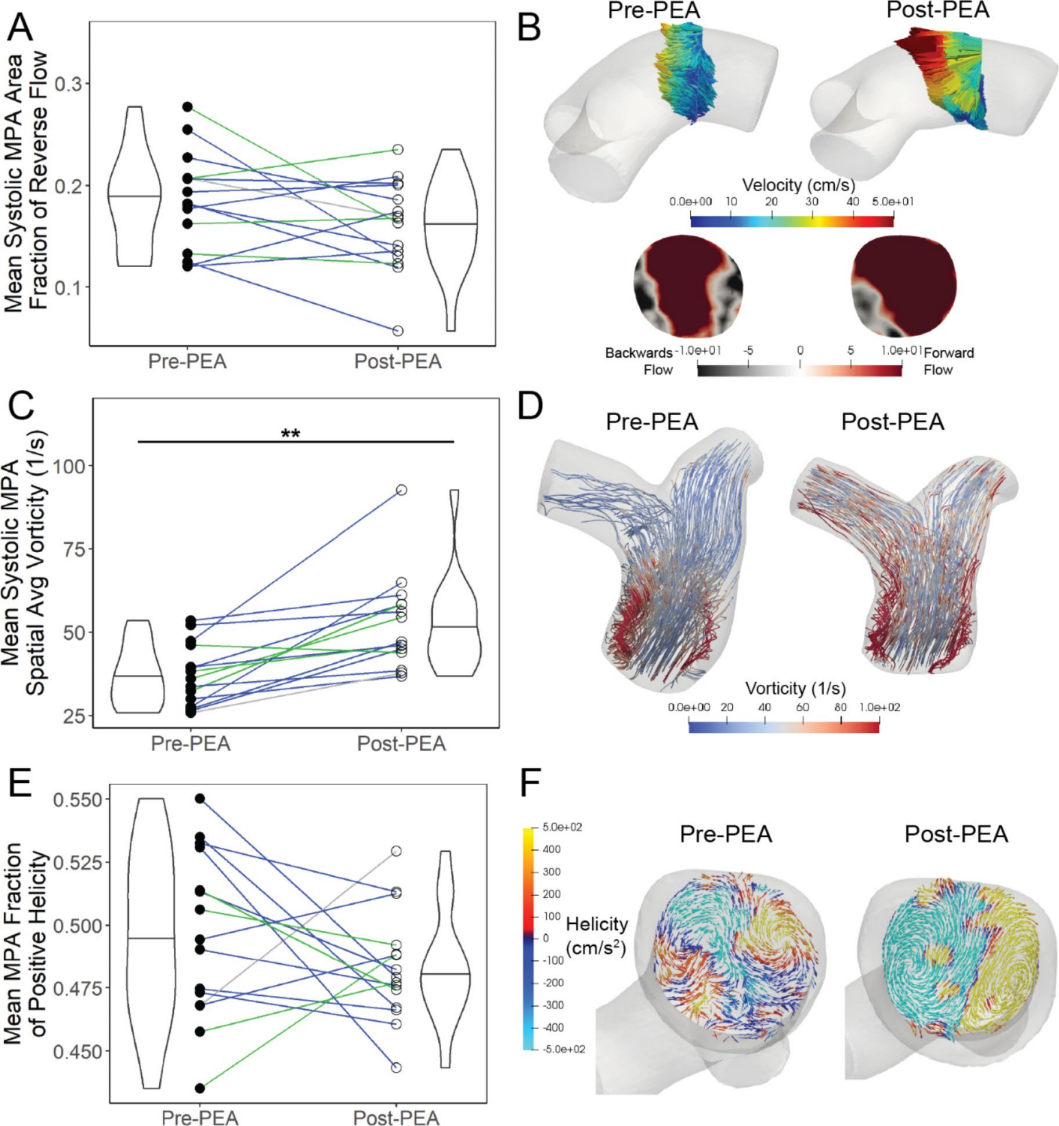
(a) The mean systolic area fraction of reverse flow in a slice in the MPA decreased slightly post-PEA. (b) A cross-sectional slice in the middle of the PA trunk taken during mid-systolic downstroke shows more reverse flow in a representative patient (PH18) pre-PEA than post-PEA. (c) The mean systolic spatially averaged vorticity in the MPA increased post-PEA. (d) Visually this is shown in a representative patient (PH7) pre- and post-PEA with streamlines colored by vorticity during peak vorticity. (e) The mean fraction of positive helicity was approximately half of the MPA volume for the pre/post-PEA group. (f) A cross-sectional MPA slice taken during systole with surface vectors projecting forward moving velocity onto the slice and colored by helicity shows distinct counter rotating structures in the post-PEA (PH18) condition as opposed to the pre-PEA condition. Colored lines (a, c, & e) represent the San Diego classification of the PEA specimens for each patient –blue = Type II, green = Type III, grey = not recorded. *=p < 0.05, **=p < 0.01

The mean systolic MPA area fraction of reverse flow decreased from pre-PEA to post-PEA (Fig. 5a). The typical decrease in area fraction of reverse flow is shown in Fig. 5b with a representative patient pre- and post-PEA. Area fraction of reverse flow was weakly correlated with both volume (r = 0.24, P = 0.2) and velocity (r = -0.31, P = 0.09) for all pre-PEA and post-PEA timepoints (**Additional file 6**), which both improved significantly following a PEA.

There were distinct swirling structures of high vorticity and low velocity in all patients with CTEPH. The vorticity in the MPA, averaged spatially over the MPA volume and temporally over the systolic period, increased significantly post-PEA (Fig. 5c).

We simulated the volume and flow similar to the pre- and post-PEA conditions. We found increased vorticity with independent decreases in volume and increases in flow analogous to the vorticity trends in the 4D flow CMR data (**Additional file 1**). A correlation matrix between 4D flow-derived metrics (**Additional file 6**) showed MPA vorticity was strongly correlated with velocity (r = 0.80, P < < 0.001) and moderately correlated with volume (r = -0.60, P < 0.001) for all pre-PEA and post-PEA timepoints.

There were no significant changes in the mean MPA fraction of positive helicity (Fig. 5e) or the maximum RPA HFI from pre-PEA to post-PEA. MPA fraction of positive helicity was normally distributed and varied up to 16% and RPA HFI varied up to 30% from pre-PEA to post-PEA. The fraction of positive helicity remained at ~ 0.5 from the pre-PEA to post-PEA timepoint indicating approximately equal volumes of positive and negative helicity but with variation between patients. Similarly, relatively small changes in helicity were also found in the max RPA HFI where the largest difference between pre-PEA to post-PEA was ~ 0.1 which corresponds to a < 7° difference in the average angle between velocity and vorticity. In Fig. 5f, surface vectors showing the projection of the velocity moving in the forward direction in a cross-sectional slice of the MPA during systole illustrate types of flow rotation seen in pre-/post-PEA timepoints of a patient with CTEPH. There were two distinct counter-rotating vortices in the clockwise and counterclockwise directions with positive and negative helicity in the post-PEA example, representative of Dean vortices which develop in curved pipes.

### Correlations of 4D flow CMR PA metrics with pulmonary hemodynamic and RV remodeling changes

We focused on the associations between mPAP, TPR, and RVESV with 4D flow CMR-derived PA metrics at baseline with all pre-PEA and post-PEA timepoints as well as with longitudinal changes from pre-PEA to post-PEA in patients with CTEPH. The most prominent associations with hemodynamic and RV remodeling changes were found with longitudinal changes in MPA and RPA area, centerline velocities, and helicity metrics from pre- to post-PEA. Correlations between 4D flow-derived PA metrics are listed in **Additional file 6**.

**Fig. 6:**
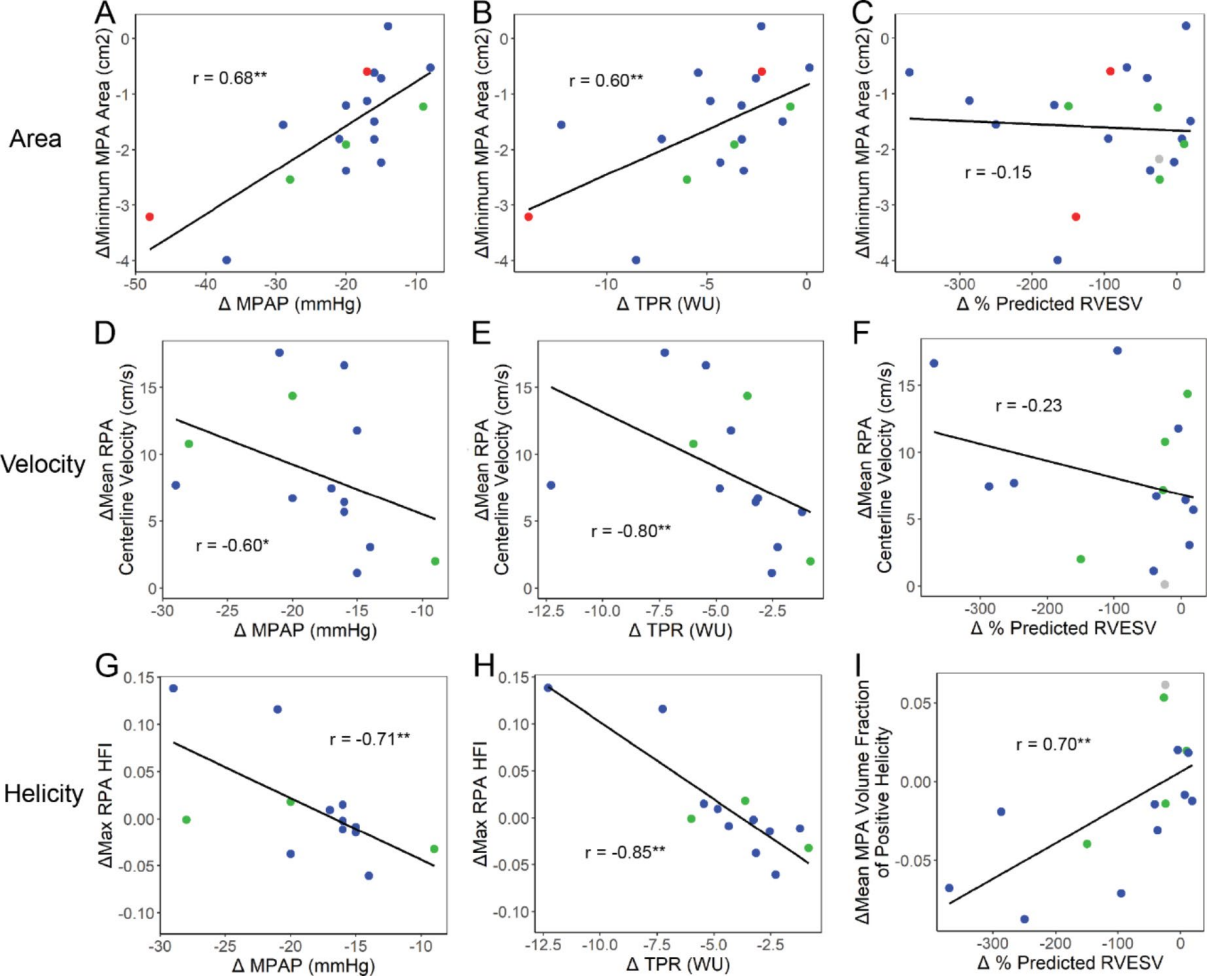
Longitudinal changes of 4D flow-derived PA area, velocity, and helicity were associated with mean PA pressure (mPAP), total pulmonary resistance (TPR), and percent predicted RV end-systolic volume (RVESV) from pre-PEA to post-PEA (negative ∆ values indicating a decrease in value from pre- to post-PEA). Decreases in minimum MPA area were associated with decreases in (a) mPAP and (b) TPR, but not with (c) % predicted RVESV. Increases in RPA centerline velocity was associated with decreases in (d) mPAP and (e) TPR, but not (f) % predicted RVESV. Increases in RPA helical flow index (HFI) were strongly associated with decreases in (g) mPAP and (h) TPR. Decreases in the MPA fraction of positive helicity were strongly associated with decreases in (i) % predicted RVESV. Correlations with area used n = 20 patients with clear anatomical resolution in the 4D flow images; velocity and helicity correlations used n = 15 patients to exclude 4D flow CMRs that did not maintain conservation of flow. Colored dots in (c)-(h) represent the San Diego classification of the PEA specimens for each patient – red = Type I, blue = Type II, green = Type III, grey = not recorded. Significance of the Spearman correlation denoted by: *=p < 0.05, **=p < 0.01

mPAP and TPR correlations with 4D flow metrics at baseline and with longitudinal changes pre-PEA to post-PEA are summarized in **Additional file 8**. At baseline, larger values of mPAP (r = -0.72, P < < 0.001) and TPR (r = -0.74, P < < 0.001) were strongly associated with larger mean RPA centerline velocities. Longitudinally, larger decreases from pre-PEA to post-PEA in mPAP and TPR were strongly associated with larger decreases in the minimum MPA Area (mPAP: r = 0.68, P = 0.002; TPR: r = 0.60, P = 0.009; Fig. 6a, b) and larger increases in the mean RPA centerline velocity (mPAP: r = -0.60, P = 0.03; TPR: r = -0.80, P = 0.002; Fig. 6d, e). Additionally, larger longitudinal decreases in mPAP and TPR following a PEA were strongly associated with larger decreases in the minimum MPA volume (mPAP: r = 0.58, P = 0.01; TPR: r = 0.67, P = 0.003). Though 4D flow-derived velocity and vorticity were moderately correlated with volume (r = -0.53 to -0.49, P < 0.002), area (r = -0.55 to -0.54, P < 0.002), and with each other (r = 0.74, P < < 0.001) at baseline, helicity metrics such as the maximum RPA HFI were not strongly correlated with other 4D flow-derived PA metrics (**Additional file 6**). Furthermore, though there was no significant difference in the maximum RPA HFI from pre- to post-PEA, longitudinal decreases from pre-PEA to post-PEA in mPAP and TPR were strongly associated with changes in the maximum RPA HFI capturing helicity intensity (mPAP: r = -0.71, P = 0.007, TPR: r = -0.85, P < 0.001, Fig. 6 g, h) following a PEA. Longitudinal increases in the max RPA HFI following a PEA were well correlated with pre- to post-PEA increases in the RPA Dean number (r = 0.77, P = 0.001, **Additional file 9**). There were weaker correlations between longitudinal changes in outcomes and LPA 4D flow derived metrics, as described in **Additional file 11**).

Percent predicted RVESV and RVEF correlations with 4D flow metrics at baseline and with longitudinal changes are listed in **Additional file 10**. At baseline, larger values of % predicted RVESV were moderately associated with larger minimum MPA volumes (r = 0.51, P = 0.001) and smaller mean systolic MPA vorticity (r = -0.58, P < < 0.001). In contrast, longitudinal changes in volumes and vorticity were not associated with changes in RVESV following a PEA. Areas and velocity were also not associated with changes in RVESV (Fig. 6c, f) in contrast with the associations with mPAP and TPR. Longitudinally, we found that larger decreases from pre-PEA to post-PEA in % predicted RVESV were strongly associated with larger decreases in the mean MPA volume fraction of positive helicity (r = 0.70, P = 0.005, Fig. 6i) indicating less clockwise rotating helical flow, and larger increases in the minimum MPA HFI (r = -0.66, P = 0.009) indicating greater helicity intensity. The longitudinal increase in the RPA Dean number following a PEA was strongly correlated with the pre- to post-PEA decrease in the MPA fraction of positive helicity (r = -0.79, P = 0.001, **Additional file 9**).

## Discussion

In this study, we developed a novel semi-automated pipeline for 4D flow analysis. Our study is among the first to describe integrated changes in 4D flow-derived PA metrics following a PEA. In addition to changes in PA volume and pulsatility, helicity emerged as a strong metric related to mPAP, TPR and RV reverse remodeling.

With a reduction in the mPAP and TPR for patients with CTEPH post-PEA, there was a corresponding improvement in the PA dynamics with decreases in volume and wall stiffness. Previous studies show decreases in mPAP were accompanied by decreases in MPA areas and volumes [33]. Analogous to these findings, Scholzel et al. found PA diameters were predictors for hemodynamic improvement following a PEA [34]. Our study confirms this relation with improvement in mPAP and TPR associated with reduction in PA volumes and areas following a PEA. In addition to volume and area changes, stiffer PA walls have been associated with severe PH disease states [29] while greater compliance, capacitance and relative area changes were associated with healthy PAs [35, 36]. Our study further confirmed this association with reduction in PA wall stiffness following a PEA, indicating that patients with CTEPH recover their vessel wall function.

Corresponding to improvements in mPAP and decreases in volume, we found increases in centerline velocity without significant increases in the mean MPA flow rate. Increased velocity following a PEA was consistent with previous CTEPH studies [4, 9, 10, 37] indicating recovery of greater PA velocities. Our study additionally highlighted the importance of velocity flow profiles in the RPA. Consistent with increasing MPA acceleration times following a PEA in Czerner et al. [10], we found increases in RPA acceleration times following a PEA which may be explained by the increase in compliance of the vessel walls. Significant increases in the RPA centerline velocity found in our study were associated with recovery of mPAP and TPR following a PEA which may be related to the importance of removing large obstructions from the right lung during a PEA.

Understanding complex secondary flow profiles in the PAs capturing helical flow may play a key role in quantifying the afterload affecting RV reverse remodeling and recovery post-PEA. Unlike Schafer et al’s metric of helicity which takes the integral sum of both negative and positive helicity over a volume, thereby canceling each other out [14], our study investigated the balance between positive and negative helicity and the overall helicity intensity through HFI. Previous studies show that equal distributions of positive and negative helicity in counter-rotating MPA helices were associated with healthy subjects [38] and were similar to symmetric Dean vortices present in a curved pipe [39]. Our findings show that larger pre- to post-PEA decreases in the fraction of MPA clockwise helicity were strongly associated with greater recoveries in the % predicted RVESV, suggesting a preference towards counterclockwise rotating helicity in the MPA. Additionally, longitudinal changes in the RPA HFI from pre- to post-PEA were strongly associated with changes in the mPAP and TPR, suggesting changes in RPA helicity intensity reflect variations in patient recovery. Larger changes in these helicity metrics were associated with greater patient recovery following a PEA. Distinguishable helical flow structures originating from the bifurcation and leading into the RPA were found in healthy subjects [40], consistent with the association of greater patient recoveries with changes in the RPA HFI following a PEA in our study. Although the paired differences between the pre- to post-PEA helicity values were not significant, the correlations between the changes in helicity with the changes in outcomes were significant. A large spread in the baseline helicity may account for this discrepancy where patient-specific changes in helicity during recovery could be associated with PA geometry changes as a result of the RV reverse remodeling and hemodynamic recovery. Large variations in these helicity metrics from pre- to post-PEA may be due to the inherent pre-PEA patient variation in pulmonary trunk curvature and correction with hemodynamic unloading following a PEA. Greater curvature and velocity in the PAs affect secondary flow structures that helicity metrics can globally capture. Given the proximity of the PAs to the RV, reduction in RV volumes post-PEA may affect the torsion and curvature of the MPA and RPA leading to changes in helicity and flow structures and the large patient variation seen in our study. Simulation of the development of Dean vortices in a curved pipe are illustrated in **Additional file 7**. Though these structures were not clearly seen in all patients with CTEPH, experimental studies of curved arteries have shown that vessel torsion and curvature can lead to less symmetry in vortices and a more dominant vortex during systolic downstroke [17]. Detailed analysis of helicity within visual secondary flow structures may be needed to understand large patient variation following a PEA. Interestingly, there were less prominent associations with 4D flow derived metrics in the LPA than the RPA. These findings highlight the importance of the RPA hemodynamics in the recovery of patients with CTEPH, which has been noted in previous studies of PH patients [4, 14]. However, future studies should investigate the impact of the LPA in relation to the RPA following a PEA in relation to geometric changes and RV reverse remodeling and improvement. The helicity correlations reported in our study suggest RV-PA functional and structural coupling may be pertinent in tracking right heart reverse remodeling with surgical treatment or other therapies.

Previous studies have shown the utility of 4D flow-derived vorticity metrics which has been associated with inefficient blood flow in patients with pulmonary [12, 14, 15, 20–23] and aortic disease [13, 16]. Although qualitative vortices and swirling has been associated with diseased states [13, 22], our study shows global quantitative metrics of vorticity increasing post-PEA in patients with CTEPH. These values of vorticity represent the amount of local rotation within the region of interest and may capture shearing between fluid layers and the vascular wall. It may not always correspond to distinct visual swirling structures shown in Fig. 5d where we visually observe slightly higher vorticity in the middle of the PA trunk in the post-PEA streamlines. Original to our study, we demonstrated that an increase in global vorticity can be explained by an increase in velocity and decrease in volume as shown by our computational fluid dynamics simulations (**Additional file 1**) of similar PA volume dilation in CTEPH. However, as our simulations show, the increase in global vorticity may not reflect the amount of visual vortical structures in the PAs due to shearing near the PA walls that are captured by global vorticity metrics. Areas of reverse flow decreased post-PEA, consistent with Kamada et al’s study of patients with CTEPH after treatment with a balloon angioplasty [11] where area fractions were averaged over the cardiac cycle and for 30 planes in the PA trunk. Though Kamada et al’s study reported higher area fractions of backwards flow, the trend in decreasing backwards flow post-intervention mirrors the results found in our study. Our study additionally validates the findings of Kamada et al. which shows how the quantitative changes in flow recirculation were associated with vortical structures and greater energy dissipation following intervention in patients with CTEPH. The decrease in the area fraction of reverse flow implies decreased recirculation post-PEA during systole which is associated with more efficient forward laminar flow with recovery from CTEPH. However, it does not capture the true vortical structures that dynamically evolve throughout the PAs. Future studies should evaluate quantitative 4D flow metrics to automatically capture changes in vortices with robust accuracy. Though previous studies have used visual analyses to identify vortices and measure their duration [22], this can be tedious and prone to human error; automatic detection of vortical structures remains a challenge. Our attempt at defining vortex structures using Q-criterion (Q > 0) and low velocity thresholding (< 30 cm/s) resulted in a non-robust identification of isolated vortex volumes (**Additional file 12**). Since these vortical structures are especially apparent in aneurysmal cases in the PAs and aorta [21, 22, 41], improved quantitative detection of vortical and swirling flow structures should be developed. Future studies could explore the utility of these quantitative 4D flow derived metrics in other pulmonary disease applications.

This study shows the potential of 4D flow-derived metrics to non-invasively evaluate recovery post-PEA, especially RV reverse remodeling. Along with standard clinical mid-term outcomes, 4D flow-derived metrics were found to improve post-PEA. Longitudinal changes in 4D flow-derived metrics of the RPA and helicity were strongly associated with recovery in PA hemodynamics and RV reverse remodeling following a PEA. The 4D flow-derived metrics reported in this study may be developed further to characterize RV remodeling and for pre-PEA risk stratification and post-PEA evaluations complementing conventional endpoints.

## Limitations

Our study has several limitations. While we were able to follow 20 patients with CTEPH before and > 6 months after a PEA, the cohort size remained small due to difficulties in obtaining mid-term follow-up visits from patients with CTEPH. Though our findings show promising utility of quantitative 4D flow-derived metrics associated with PA hemodynamic recovery and RV reverse remodeling post-PEA, larger cohorts would be needed to validate the findings presented in this study. Furthermore, an age and sex-matched control group with the same CMR sequence as the CTEPH patients is needed for comparison to the CTEPH patient cohort. Although control patients with normal PA hemodynamics and right heart function were included in Additional Files 13–17, these patients had mild or moderate aortic regurgitation and was limited to 8 subjects that were not sex matched. Increased sample sizes in the control group with sex-matched subjects would be needed to further evaluate the utility of 4D flow in a variety of disease states and eliminate biases in sex. Consideration of the specimens removed, either large proximal or distal obstructions, during the PEA of each patient with CTEPH will be needed to precisely correlate recovery with surgical outcomes in a larger cohort. Additionally, the influence of PH medications before and following a PEA was not included in this study and could be important to discern the effects on the flow profiles independent of the PEA.

The semi-automated processing pipeline to extract velocity and anatomic metrics from the 4D flow images allowed faster gathering of information on a patient-specific basis, but the segmentations and corrections needed to obtain an accurate PA anatomy were tedious. Though there are many automated image segmentation tools developed for medical images, most rely on very high-resolution images from CT scans or CMR angiograms. Because 4D flow CMR has a lower spatial resolution and the PAs are surrounded by the heart and other vessels that were also illuminated by contrast, current automated image segmentation methods of the PAs were limited and had to be corrected manually. To obtain fast processing of 4D flow velocity metrics, improved automated image segmentation is needed to reduce the time and manual processing of the anatomy. Furthermore, the image segmentation used in this study was based on 4D flow which was unable to precisely capture proximal obstructions and wall thickness where there was limited blood flow. Thus, future anatomic metrics should use CT scans to analyze these metrics and to capture those obstructions more precisely, especially to determine areas where obstructions were unable to be removed.

## Conclusion

We investigated longitudinal 4D flow CMR metrics of the PAs to characterize recovery and RV reverse remodeling following a PEA in patients with CTEPH. Our study proposes a novel pipeline for 4D flow analysis of pulmonary hemodynamics. Our findings demonstrate the hemodynamic trajectory of PA and RV recovery following a PEA. 4D flow-derived PA metrics further highlight the importance of helicity and RPA flow dynamics to evaluate longitudinal reverse remodeling of the RV and recovery in patients with CTEPH following a PEA.

## Electronic supplementary material

Below is the link to the electronic supplementary material.

Supplementary Material 1

Supplementary Material 2

Supplementary Material 3

Supplementary Material 4

Supplementary Material 5

Supplementary Material 6

Supplementary Material 7

Supplementary Material 8

Supplementary Material 9

Supplementary Material 10

Supplementary Material 11

Supplementary Material 12

Supplementary Material 13

Supplementary Material 14

Supplementary Material 15

Supplementary Material 16

Supplementary Material 17

## Data Availability

The datasets used and analyzed during the current study are available from the corresponding author on reasonable request. The 4D flow post-processing pipeline is publicly available at: https://github.com/SimVascular/Tools/tree/master/PostProcessingPipeline_4DFlow.

## List of abbreviations

4D flow CMR: Four-dimensional flow cardiovascular magnetic resonance
bSSFP: Balanced steady-state free precession
CMR: Cardiovascular magnetic resonance
CTEPH: Chronic thromboembolic pulmonary hypertension
De: Dean number
ECG: Electrocardiogram
H_*d*_: Helical density
HFI: Helical flow index
LPA: Left pulmonary artery
LV: Left ventricle/left ventricular
LVEF: Left ventricular ejection fraction
MPA: Main pulmonary artery
mPAP: Mean pulmonary artery pressure
PA: Pulmonary artery
PEA: Pulmonary endarterectomy
PH: Pulmonary hypertension
RAC: Relative area change
RHC: Right heart catheterization
RPA: Right pulmonary artery
RV: Right ventricle/right ventricular
RVEDV: Right ventricular end-diastolic volume
RVEDVI: Right ventricular end-diastolic volume index
RVEF: Right ventricular ejection fraction
RVESV: Right ventricular end-systolic volume
RVESVI: Right ventricular end-systolic volume index
RVSV: Right ventricular stroke volume
TPR: Total pulmonary resistance
VENC: Velocity encoding
