# Supplementary material for: 4D flow cardiovascular magnetic resonance recovery profiles following pulmonary endarterectomy in chronic thromboembolic pulmonary hypertension
Source: J Cardiovasc Magn Reson. 2022 Nov 14;24:59. doi: 10.1186/s12968-022-00893-x (PMC9661778; doi:10.1186/s12968-022-00893-x)
Supplement: Supplementary file 1 — Supplementary Material 1 [file 12968_2022_893_MOESM1_ESM.docx]

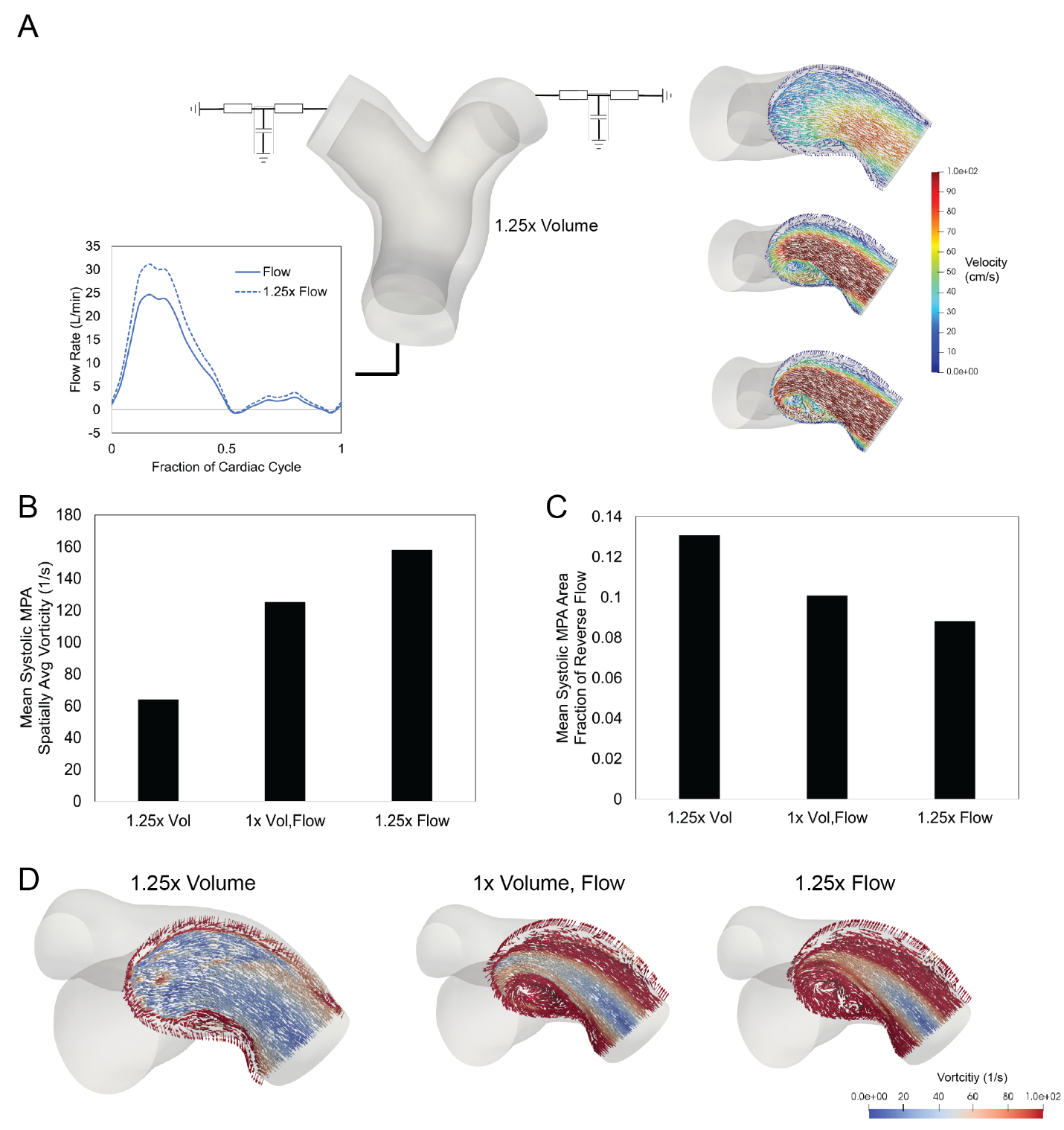


**Additional file 1:** (A) Computational fluid dynamics simulations modeling of the PAs with independent changes in 1.25x Volume (representing pre-PEA conditions) and 1.25X Flow (representing a variation in post-PEA conditions) with an imposed inflow waveform and 3-element Windkessel boundary conditions from the PH2 post-PEA anatomy show (b) increasing spatially averaged MPA vorticity during systole from pre-PEA to post-PEA, (c) decreasing area fractions of reverse flow, and (d) visual increases in vorticity with smaller volumes and flows, especially near the walls where shearing occurs.
