# Supplementary material for: 4D flow cardiovascular magnetic resonance recovery profiles following pulmonary endarterectomy in chronic thromboembolic pulmonary hypertension
Source: J Cardiovasc Magn Reson. 2022 Nov 14;24:59. doi: 10.1186/s12968-022-00893-x (PMC9661778; doi:10.1186/s12968-022-00893-x)
Supplement: Supplementary file 2 — Supplementary Material 2 [file 12968_2022_893_MOESM2_ESM.docx]

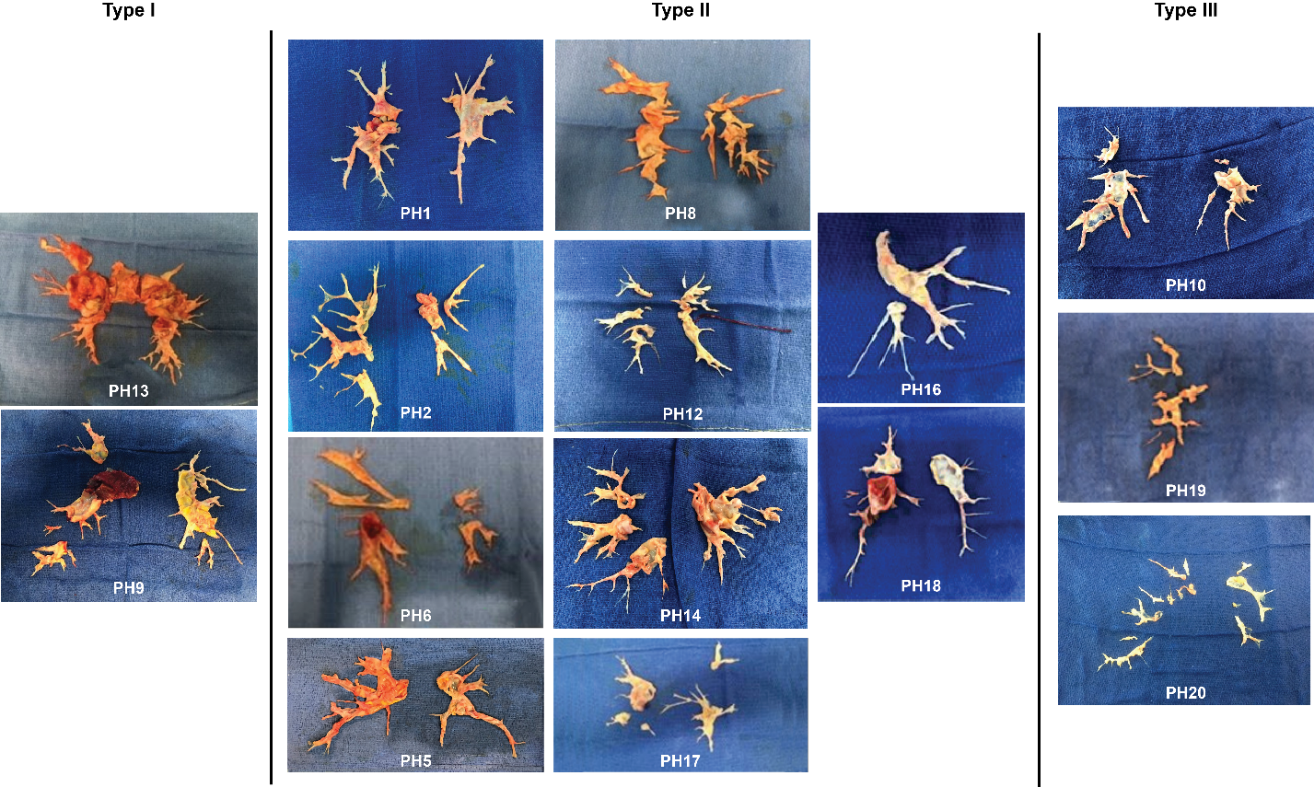


**Additional File 2:** San Diego classification of pulmonary endarterectomy specimens removed from patients with CTEPH during surgical procedure. Not all specimen pictures are displayed, but the visual distribution of type I, type II, and type III specimens approximately represents the distribution for all patient specimens.
