# Supplementary material for: 4D flow cardiovascular magnetic resonance recovery profiles following pulmonary endarterectomy in chronic thromboembolic pulmonary hypertension
Source: J Cardiovasc Magn Reson. 2022 Nov 14;24:59. doi: 10.1186/s12968-022-00893-x (PMC9661778; doi:10.1186/s12968-022-00893-x)
Supplement: Supplementary file 3 — Supplementary Material 3 [file 12968_2022_893_MOESM3_ESM.docx]

**Additional file 3:** Longitudinal Patient Outcomes

|  | CTEPH, Pre-PEA (n=20) | CTEPH, Post-PEA (n=20) | Control (n=8) |
| --- | --- | --- | --- |
| 4D flow HR (bpm)^†‡‡^ | 77 +/- 10 | 80 +/- 12 | 69 +/- 7 |
| MPAP (mmHg)** | 44 +/- 12  (n=18) | 24 +/- 5  (n=18) |  |
| TPR (WU)** | 9.0 +/- 4.3  (n=18) | 4.3 +/- 1.7  (n=18) |  |
| 6-MWTD (m)* | 407 +/- 164  (n=12) | 479 +/- 105  (n=12) |  |
| NYHA Class** | I=1 (5%)  II=8 (40%)  III=7 (35%)  IV=1 (5%)  Not recorded=3 (15%) | I=7 (35%)  II=9 (45%)  III=1 (5%)  IV=0 (0%)  Not recorded=3 (15%) |  |
| % Predicted RVEDV** | 122 +/- 53 | 93 +/- 33 | 100 +/- 34 |
| % Predicted RVESV**^††^ | 258 +/- 170 | 157 +/- 77 | 144 +/- 47 |
| % Predicted RVEF**^††^ | 60 +/- 19 | 75 +/- 13 | 85 +/- 10 |

CTEPH Pre-PEA vs Post-PEA significance determined from a paired t-test. Percent predicted right ventricle (RV) metrics adjusted for age, sex, weight, and height using normative equations (Kawut 2011). CTEPH Pre/Post-PEA vs Control significance determined from Welch’s t-test. Significant differences in the NYHA Class were computed using the Wilcoxon signed-rank test. Significance between groups denoted by: *=p<0.05 Pre-/Post-PEA, **=p<0.01 Pre-/Post-PEA, ^†^=p<0.05 Pre-PEA/Control, ^††^=p<0.01 Pre-/Control, ^‡^=p<0.05 Post-PEA/Control, ^‡‡^=p<0.01 Post-PEA/Control

Values are mean ± standard deviation or n (%). HR, heart rate; RVESV, right ventricular end-systolic volume; RVEDV, right ventricular end-diastolic volume; RVEF, right ventricular ejection fraction; MPAP, mean pulmonary artery pressure; TPR, total pulmonary resistance; PEA, pulmonary endarterectomy; 6-MWTD, 6-minute walk test distance; NYHA, New York Heart Association
