# Supplementary material for: 4D flow cardiovascular magnetic resonance recovery profiles following pulmonary endarterectomy in chronic thromboembolic pulmonary hypertension
Source: J Cardiovasc Magn Reson. 2022 Nov 14;24:59. doi: 10.1186/s12968-022-00893-x (PMC9661778; doi:10.1186/s12968-022-00893-x)
Supplement: Supplementary file 4 — Supplementary Material 4 [file 12968_2022_893_MOESM4_ESM.docx]

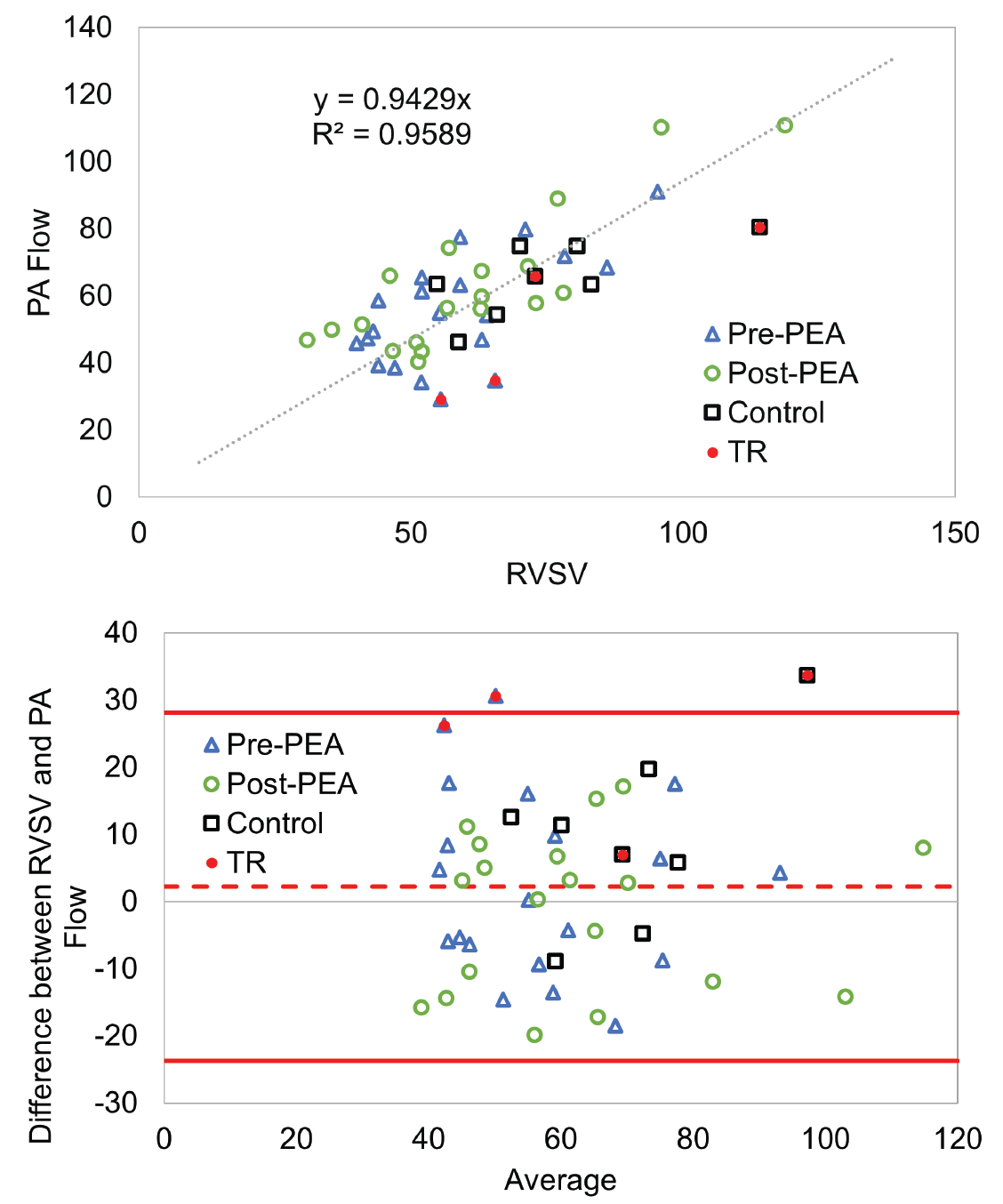


**Additional file 4:** Right ventricular stroke volume (RVSV) was compared against 4DMR-derived pulmonary artery (PA) flow volume taken at a cross-sectional slice 10% down the length of the MPA to validate measurements. There were strong correlations between RVSV and PA flow. A Bland-Altman plot shows fairly good agreement within 20 ml between RVSV and PA flow with some extreme differences occurring due to tricuspid regurgitation (TR). Red lines on the Bland-Altman plot indicate the lower and upper IQR of -24 and 28, respectively. Blue triangle=Pre-PEA, green circle=Post-PEA, black square=Control, red overlayed circle=presence of moderate to severe tricuspid regurgitation.
