# Supplementary material for: 4D flow cardiovascular magnetic resonance recovery profiles following pulmonary endarterectomy in chronic thromboembolic pulmonary hypertension
Source: J Cardiovasc Magn Reson. 2022 Nov 14;24:59. doi: 10.1186/s12968-022-00893-x (PMC9661778; doi:10.1186/s12968-022-00893-x)
Supplement: Supplementary file 5 — Supplementary Material 5 [file 12968_2022_893_MOESM5_ESM.docx]

**Additional file 5:** Summary of 4D flow-derived metrics for Pre-, Post-PEA, and Control groups

|  | Pre-PEA | Post-PEA | Control |
| --- | --- | --- | --- |
| Mean MPA Volume (ml) ^**††‡‡^ | 66 ± 23  (n=20) | 52 ± 20  (n=20) | 54 ± 24 |
| Mean LPA Volume (ml) ^**††^ | 11 ± 4  (n=20) | 9 ± 4  (n=20) | 9 ± 4 |
| Mean RPA Volume (ml) ^**††‡‡^ | 15 ± 4  (n=20) | 12 ± 5  (n=20) | 13 ± 5 |
| MPA RAC^**^ | 0.11 ± 0.08  (n=20) | 0.17 ± 0.07  (n=20) | 0.14 ± 0.08 |
| Compliance (cm^2^/mmHg)^**^ | 0.019 ± 0.013  (n=12) | 0.052 ± 0.036  (n=12) |  |
| Distensibility (%/mmHg)** | 0.21 ± 0.15  (n=12) | 0.66 ± 0.47  (n=12) |  |
| Mean MPA Flow Rate (L/min) | 4.7 ± 1.6  (n=15) | 5.3 ± 1.4  (n=15) | 4.9 ±1.4 |
| Mean LPA Flow Rate (L/min) | 2.3 ± 1.2  (n=15) | 2.0 ±0.7  (n=15) | 2.1 ±0.9 |
| Mean RPA Flow Rate (L/min)^*‡‡^ | 2.4 ± 1.0  (n=15) | 3.2 ±0.9  (n=15) | 2.7 ± 0.9 |
| Max MPA Centerline Velocity (cm/s)^**††^ | 56 ± 14  (n=15) | 76 ± 21  (n=15) | 70 ± 21 |
| Max LPA Centerline Velocity (cm/s)^**††^ | 31 ± 14  (n=15) | 45 ± 15  (n=15) | 41 ± 16 |
| Max RPA Centerline Velocity (cm/s)^**††^ | 30 ± 10  (n=15) | 52 ± 12  (n=15) | 46 ± 18 |
| RPA Acceleration Time Ratio^*^ | 0.12 ± 0.03  (n=15) | 0.16 ± 0.05  (n=15) | 0.15 ± 0.04 |
| Mean Systolic MPA Spatially Averaged Vorticity (1/s)^**††^ | 37 ± 9  (n=15) | 52 ± 14  (n=15) | 45 ± 13 |
| Mean Systolic MPA Area Fraction of Reverse Flow^††‡‡^ | 0.19 ± 0.05  (n=15) | 0.16 ± 0.05  (n=15) | 0.15 ± 0.06 |
| Mean Systolic LPA Area Fraction of Reverse Flow*^‡‡^ | 0.10 ± 0.07  (n=15) | 0.14 ± 0.07  (n=15) | 0.06 ± 0.05 |
| Mean MPA Volume Fraction of Positive Helicity | 0.50 ± 0.03  (n=15) | 0.48 ± 0.02  (n=15) | 0.49 ± 0.03 |
| Min MPA Spatially Averaged HFI | 0.41 ± 0.03 | 0.41 ± 0.02 | 0.40 ± 0.03 |
| Max RPA Spatially Averaged HFI^†‡^ | 0.46 ± 0.05  (n=15) | 0.46 ± 0.04  (n=15) | 0.47 ±0.04 |
| RVEDVI (ml/m^2^)^**^ | 82 +/- 35  (n=20) | 63 +/- 25  (n=20) | 73 +/- 17 |
| RVESVI (ml/m^2^)^**††^ | 51 +/- 32  (n=20) | 31 +/- 15  (n=20) | 33 +/- 6 |
| RVEF (%)^**††^ | 42 +/- 13  (n=20) | 52 +/- 9  (n=20) | 58 +/- 7 |
| RVSV (ml) | 58 +/- 16  (n=20) | 62 +/- 21  (n=20) | 75 +/- 19 |

CTEPH Pre-PEA vs Post-PEA significance determined from a paired t-test. CTEPH Pre/Post-PEA vs Control significance determined from Welch’s t-test. Values are mean ± standard deviation (n=sample size). Significance between groups denoted by: *=p<0.05 Pre-/Post-PEA, **=p<0.01 Pre-/Post-PEA, ^†^=p<0.05 Pre-PEA/Control, ^††^=p<0.01 Pre-/Control, ^‡^=p<0.05 Post-PEA/Control, ^‡‡^=p<0.01 Post-PEA/Control
