# Supplementary material for: 4D flow cardiovascular magnetic resonance recovery profiles following pulmonary endarterectomy in chronic thromboembolic pulmonary hypertension
Source: J Cardiovasc Magn Reson. 2022 Nov 14;24:59. doi: 10.1186/s12968-022-00893-x (PMC9661778; doi:10.1186/s12968-022-00893-x)
Supplement: Supplementary file 6 — Supplementary Material 6 [file 12968_2022_893_MOESM6_ESM.docx]

**Additional file 6:** Correlations between 4D flow-derived PA metrics from patients with CTEPH at pre- and post-PEA timepoints

|  |  | **PA Volumetric Data** | | **Velocity Flow Profiles** | | | **Secondary Flow Profiles** | | | | |
| --- | --- | --- | --- | --- | --- | --- | --- | --- | --- | --- | --- |
|  |  | Min MPA Area | MPA RAC | Mean MPA Flow | Mean MPA Center-line Velocity | Mean RPA Center-line Velocity | Mean Systolic MPA Spatial Avg Vorticity | Mean Systolic MPA Area Fraction of Re-  verse Flow | Mean MPA Fraction of Positive Helicity | Min MPA Spatial Avg HFI | Max RPA Spatial Avg HFI |
| **PA Volumetric Data** | Min MPA Volume | 0.92 | -0.44 | 0.05 | -0.26 | -0.49 | -0.60 | 0.24 | 0.10 | -0.19 | 0.09 |
|  | Min MPA Area |  | -0.34 | 0.10 | -0.23 | -0.48 | -0.65 | 0.31 | 0.14 | -0.04 | 0.12 |
|  | MPA RAC |  |  | -0.06 | -0.06 | 0.31 | 0.19 | -0.04 | -0.26 | 0.39 | -0.32 |
| **Velocity Flow Profiles** | Mean MPA Flow |  |  |  | 0.81 | 0.60 | 0.44 | -0.33 | -0.27 | -0.05 | 0.43 |
|  | Mean MPA Centerline Velocity |  |  |  |  | 0.79 | 0.80 | -0.31 | -0.26 | -0.10 | 0.42 |
|  | Mean RPA Centerline Velocity |  |  |  |  |  | 0.84 | -0.41 | -0.29 | 0.07 | 0.16 |
| **Secondary Flow Profiles** | Mean Systolic MPA Spatial Avg Vorticity |  |  |  |  |  |  | -0.28 | -0.23 | 0.00 | 0.12 |
|  | Mean Systolic MPA Area Fraction of Reverse Flow |  |  |  |  |  |  |  | 0.20 | -0.21 | -0.18 |
|  | Mean MPA Fraction of Positive Helicity |  |  |  |  |  |  |  |  | -0.04 | -0.12 |
|  | Min MPA Spatial Avg HFI |  |  |  |  |  |  |  |  |  | 0.11 |

Correlations indicated by Spearman’s rho-value. HFI=helical flow index
