# Supplementary material for: 4D flow cardiovascular magnetic resonance recovery profiles following pulmonary endarterectomy in chronic thromboembolic pulmonary hypertension
Source: J Cardiovasc Magn Reson. 2022 Nov 14;24:59. doi: 10.1186/s12968-022-00893-x (PMC9661778; doi:10.1186/s12968-022-00893-x)
Supplement: Supplementary file 7 — Supplementary Material 7 [file 12968_2022_893_MOESM7_ESM.docx]

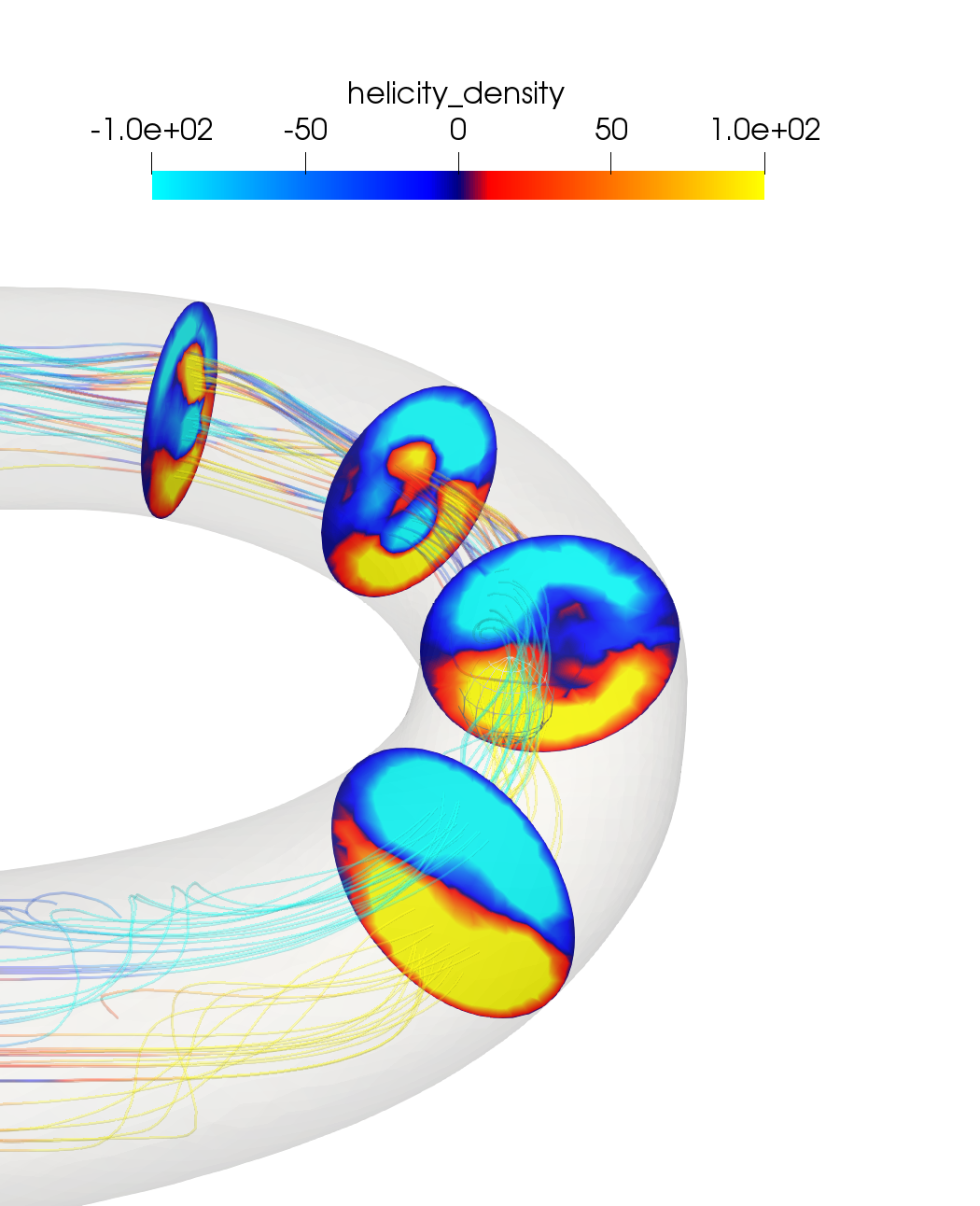


**Additional file 7:** Simulation of pulsatile flow in a curved tube illustrate the formation of counter-rotating vortices, or Dean vortices, with positive (yellow) and negative (blue) helicity in the curved region. Streamlines originating from the cross-sectional slice in the middle show the positive and negative helicity throughout the curved tube.
