# Supplementary material for: 4D flow cardiovascular magnetic resonance recovery profiles following pulmonary endarterectomy in chronic thromboembolic pulmonary hypertension
Source: J Cardiovasc Magn Reson. 2022 Nov 14;24:59. doi: 10.1186/s12968-022-00893-x (PMC9661778; doi:10.1186/s12968-022-00893-x)
Supplement: Supplementary file 8 — Supplementary Material 8 [file 12968_2022_893_MOESM8_ESM.docx]

**Additional file 8:** Correlations between 4D flow MRI metrics and PA hemodynamics

|  | **PA Volumetric Data** | | | **Velocity Flow Profile** | | | **Secondary Flow Profile** | | | | |
| --- | --- | --- | --- | --- | --- | --- | --- | --- | --- | --- | --- |
|  | Min MPA Volume | Min MPA Area | MPA RAC | Mean MPA Flow | Mean MPA Center-line Velocity | Mean RPA Center-line Velocity | Mean Systolic MPA Spatial Avg Vorticity | Mean Systolic MPA Area Fraction of Re-verse Flow | Mean MPA Fraction of Positive Helicity | Min MPA Spatial Avg HFI | Max RPA Spatial Avg HFI |
| **Baseline Pre-PEA, Post-PEA** | | | | | | | | | | | |
| MPAP | 0.45 | 0.44 | -0.53 | -0.27 | -0.44 | -0.72 | -0.58 | 0.26 | 0.35 | -0.10 | 0.26 |
| TPR | 0.42 | 0.36 | -0.42 | -0.46 | -0.62 | -0.74 | -0.62 | 0.22 | 0.30 | -0.16 | 0.08 |
| **Longitudinal Changes from Pre-PEA to Post-PEA** | | | | | | | | | | | |
| ∆MPAP | 0.58 | 0.68 | -0.14 | -0.30 | -0.14 | -0.60 | -0.49 | 0.40 | 0.38 | -0.32 | -0.71 |
| ∆TPR | 0.67 | 0.60 | 0.09 | -0.58 | -0.58 | -0.80 | -0.69 | 0.64 | 0.40 | -0.60 | -0.85 |

Correlations indicated by Spearman’s rho-value. MPAP=Mean Pulmonary Artery Pressure, TPR=Total Pulmonary Resistance
