# Supplementary material for: 4D flow cardiovascular magnetic resonance recovery profiles following pulmonary endarterectomy in chronic thromboembolic pulmonary hypertension
Source: J Cardiovasc Magn Reson. 2022 Nov 14;24:59. doi: 10.1186/s12968-022-00893-x (PMC9661778; doi:10.1186/s12968-022-00893-x)
Supplement: Supplementary file 9 — Supplementary Material 9 [file 12968_2022_893_MOESM9_ESM.docx]

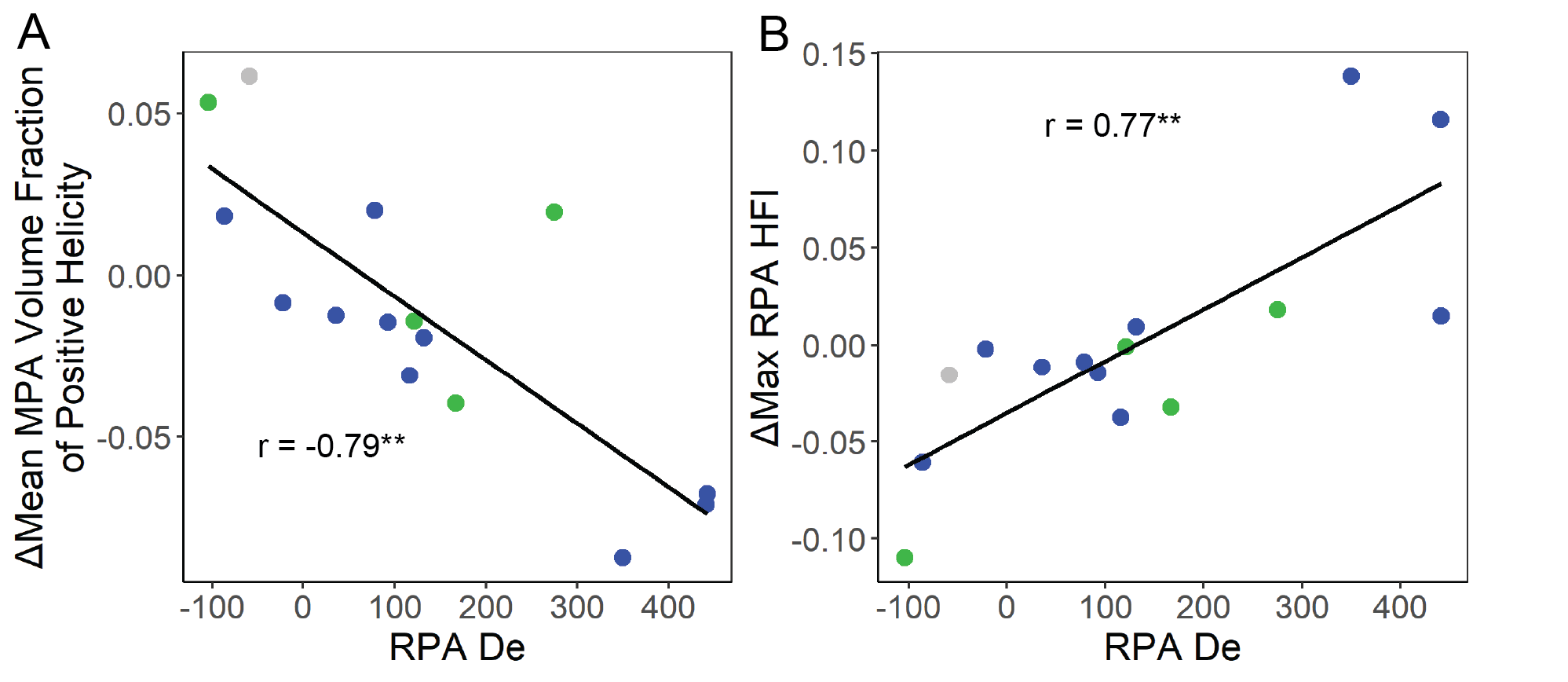


**Additional file 9**: The change in RPA Dean number from pre-PEA to post-PEA was well correlated with the (a) mean MPA volume fraction of positive helicity and (b) the max RPA spatially averaged HFI. Colored points represent the San Diego classification of the PEA specimens for each patient – red=Type I, blue=Type II, green=Type III, grey=not recorded. Significance of the Spearman correlation denoted by: *=p<0.05, **=p<0.01
