# Supplementary material for: 4D flow cardiovascular magnetic resonance recovery profiles following pulmonary endarterectomy in chronic thromboembolic pulmonary hypertension
Source: J Cardiovasc Magn Reson. 2022 Nov 14;24:59. doi: 10.1186/s12968-022-00893-x (PMC9661778; doi:10.1186/s12968-022-00893-x)
Supplement: Supplementary file 10 — Supplementary Material 10 [file 12968_2022_893_MOESM10_ESM.docx]

**Additional file 10:** Correlation between 4D flow MRI metrics and RV metrics from patients with CTEPH

|  | **PA Volumetric Data** | | | **Velocity Flow Profile** | | | **Secondary Flow Profile** | | | | |
| --- | --- | --- | --- | --- | --- | --- | --- | --- | --- | --- | --- |
|  | Min MPA Volume | Min MPA Area | MPA RAC | Mean MPA Flow | Mean MPA Center-line Velocity | Mean RPA Center-line Velocity | Mean Systolic MPA Spatial Avg Vorticity | Mean Systolic MPA Area Fraction of Re-verse Flow | Mean MPA Fraction of Positive Helicity | Min MPA Spatial Avg HFI | Max RPA Spatial Avg HFI |
| **Baseline Pre-PEA, Post-PEA, & Control** | | | | | | | | | | | |
| % Predicted RVESV | 0.51 | 0.47 | -0.27 | -0.27 | -0.43 | -0.44 | -0.58 | 0.17 | 0.45 | -0.06 | -0.14 |
| % Predicted RVEF | -0.47 | -0.45 | 0.30 | 0.35 | 0.52 | 0.53 | 0.63 | -0.24 | -0.58 | 0.07 | 0.22 |
| **Longitudinal Changes from Pre-PEA to Post-PEA** | | | | | | | | | | | |
| ∆ % Predicted RVESV | 0.04 | -0.15 | -0.20 | -0.02 | -0.04 | -0.23 | -0.17 | -0.08 | 0.70 | -0.66 | -0.31 |
| ∆ % Predicted RVEF | -0.17 | 0.06 | 0.14 | 0.35 | 0.18 | 0.37 | 0.28 | -0.09 | -0.66 | 0.36 | 0.43 |

Correlations indicated by Spearman’s rho-value. RVESV=Right Ventricle End-Systolic Volume, RVEF=Right Ventricle Ejection Fraction
