# Supplementary material for: 4D flow cardiovascular magnetic resonance recovery profiles following pulmonary endarterectomy in chronic thromboembolic pulmonary hypertension
Source: J Cardiovasc Magn Reson. 2022 Nov 14;24:59. doi: 10.1186/s12968-022-00893-x (PMC9661778; doi:10.1186/s12968-022-00893-x)
Supplement: Supplementary file 11 — Supplementary Material 11 [file 12968_2022_893_MOESM11_ESM.docx]

**Additional file 11:** Correlation between longitudinal changes in 4D flow MRI left PA metrics and outcomes

|  | Mean LPA centerline velocity | Mean Systolic LPA Area Fraction of Reverse Flow | Mean LPA Spatial Avg Vorticity | Mean Systolic LPA Spatial Avg HFI |
| --- | --- | --- | --- | --- |
| ∆ MPAP | -0.18 | 0.36 | -0.43 | 0.10 |
| ∆ TPR | -0.38 | 0.57 | -0.54 | -0.26 |
| ∆ % Predicted RVESV | 0.12 | -0.05 | 0.08 | -0.45 |
| ∆ % Predicted RVEF | 0.25 | -0.41 | 0.03 | 0.28 |
