# Supplementary material for: 4D flow cardiovascular magnetic resonance recovery profiles following pulmonary endarterectomy in chronic thromboembolic pulmonary hypertension
Source: J Cardiovasc Magn Reson. 2022 Nov 14;24:59. doi: 10.1186/s12968-022-00893-x (PMC9661778; doi:10.1186/s12968-022-00893-x)
Supplement: Supplementary file 12 — Supplementary Material 12 [file 12968_2022_893_MOESM12_ESM.docx]

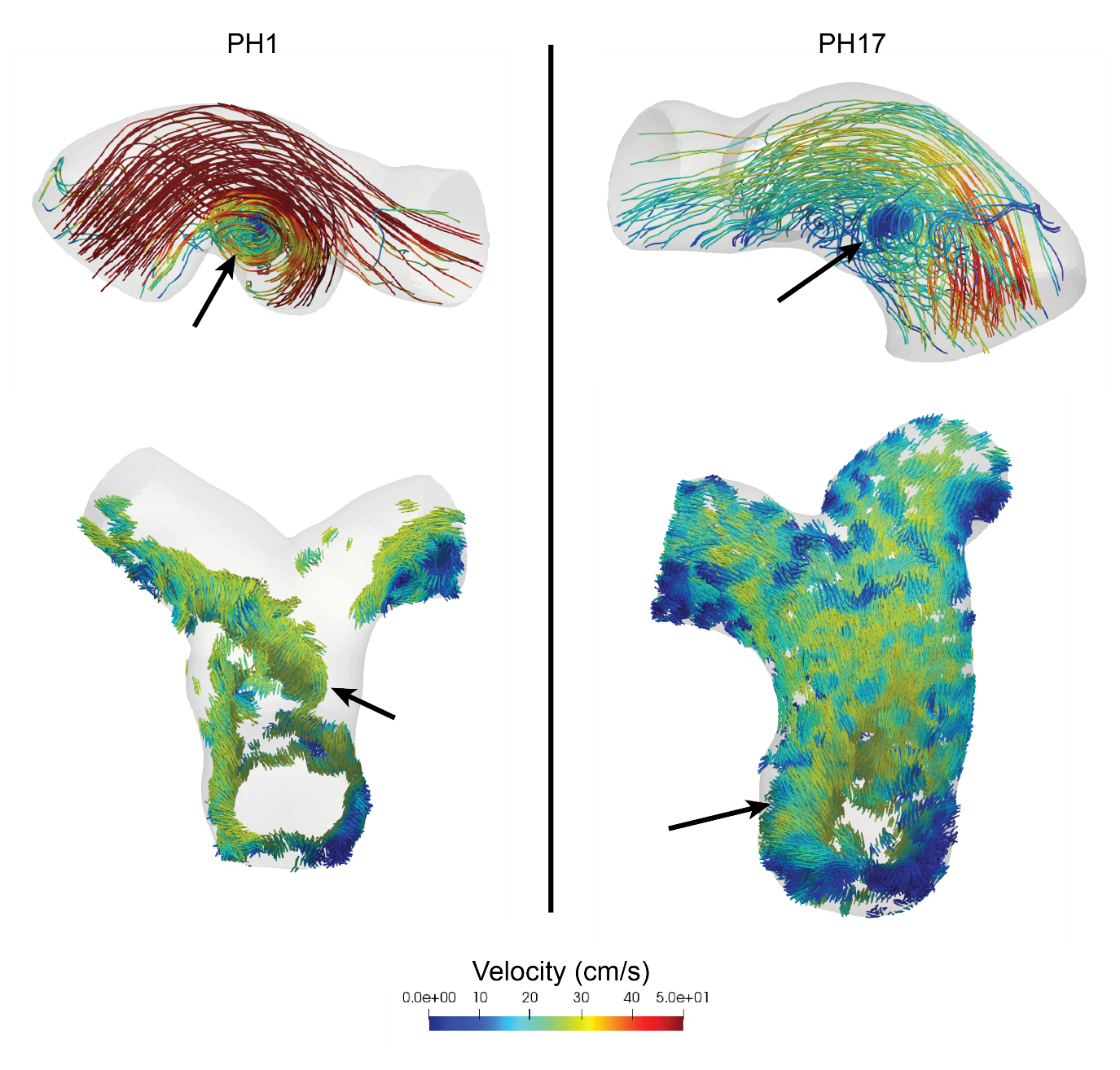


**Additional file 12**: Streamlines of two patients with CTEPH, PH1 and PH17, post-PEA show distinct swirling structures in the bifurcation and along the right side of the pulmonary trunk, respectively, denoted by arrows. Isolation of the volumes defining these swirling structures were attempted using thresholds of Q>0 and velocity<30cm/s. These thresholds worked well in PH1 but not in PH17 likely due to the lower velocity present in PH17.
