# Supplementary material for: 4D flow cardiovascular magnetic resonance recovery profiles following pulmonary endarterectomy in chronic thromboembolic pulmonary hypertension
Source: J Cardiovasc Magn Reson. 2022 Nov 14;24:59. doi: 10.1186/s12968-022-00893-x (PMC9661778; doi:10.1186/s12968-022-00893-x)
Supplement: Supplementary file 13 — Supplementary Material 13 [file 12968_2022_893_MOESM13_ESM.docx]

**Additional file 13:** We also recruited 8 patients referred for a clinical MRI due to aortic regurgitation as part of a control group. These patients had no evidence of PH, RV systolic dysfunction (RVEF>50%), LV systolic dysfunction (LVEF>50%), or severe valvular disease. Though these patients were referred for mild to moderate aortic regurgitation, they had normal PA hemodynamics and right heart function. For the control patients, a different CMR protocols was performed on a 3 T Discovery MR 750W CMR system (General Electric Healthcare, Waukesha, WI, USA) with a 32-channel coil for cardiac imaging. Similarly, respiratory-gated and cardiac triggered 4D flow MRI data were acquired in a 3D coronal volume with a Venc of 300-500 cm/s to capture valvular regurgitation, 30 cardiac frames acquired per cardiac cycle, and a spatial resolution of 1.9 x 1.9 x 2.2 mm^3^. All 4D flow MRI sequences were acquired after biphasic injection of 0.4 ml/kg of Gadoretic acid (Dotarem, Guerbet, France) or 0.2 ml/kg of Gadobutrol (Gadovist, Bayer, Germany), including a bolus followed by a slow infusion at a rate of 0.1 ml/s. Fisher’s exact test was used to determine significance in categorical variables between patients with CTEPH and the control group. There were significant differences in the MPA and RPA volumes between post-PEA patients and the control group. However, the venc in the 4D flow sequence for the control patients was higher than the patients with CTEPH which may cause differences in the comparison for low velocity flows. Though we did not directly compare low velocity flows in our analysis, this may be an important comparison in future studies of secondary PA flow profiles.
