# Supplementary material for: 4D flow cardiovascular magnetic resonance recovery profiles following pulmonary endarterectomy in chronic thromboembolic pulmonary hypertension
Source: J Cardiovasc Magn Reson. 2022 Nov 14;24:59. doi: 10.1186/s12968-022-00893-x (PMC9661778; doi:10.1186/s12968-022-00893-x)
Supplement: Supplementary file 15 — Supplementary Material 15 [file 12968_2022_893_MOESM15_ESM.docx]

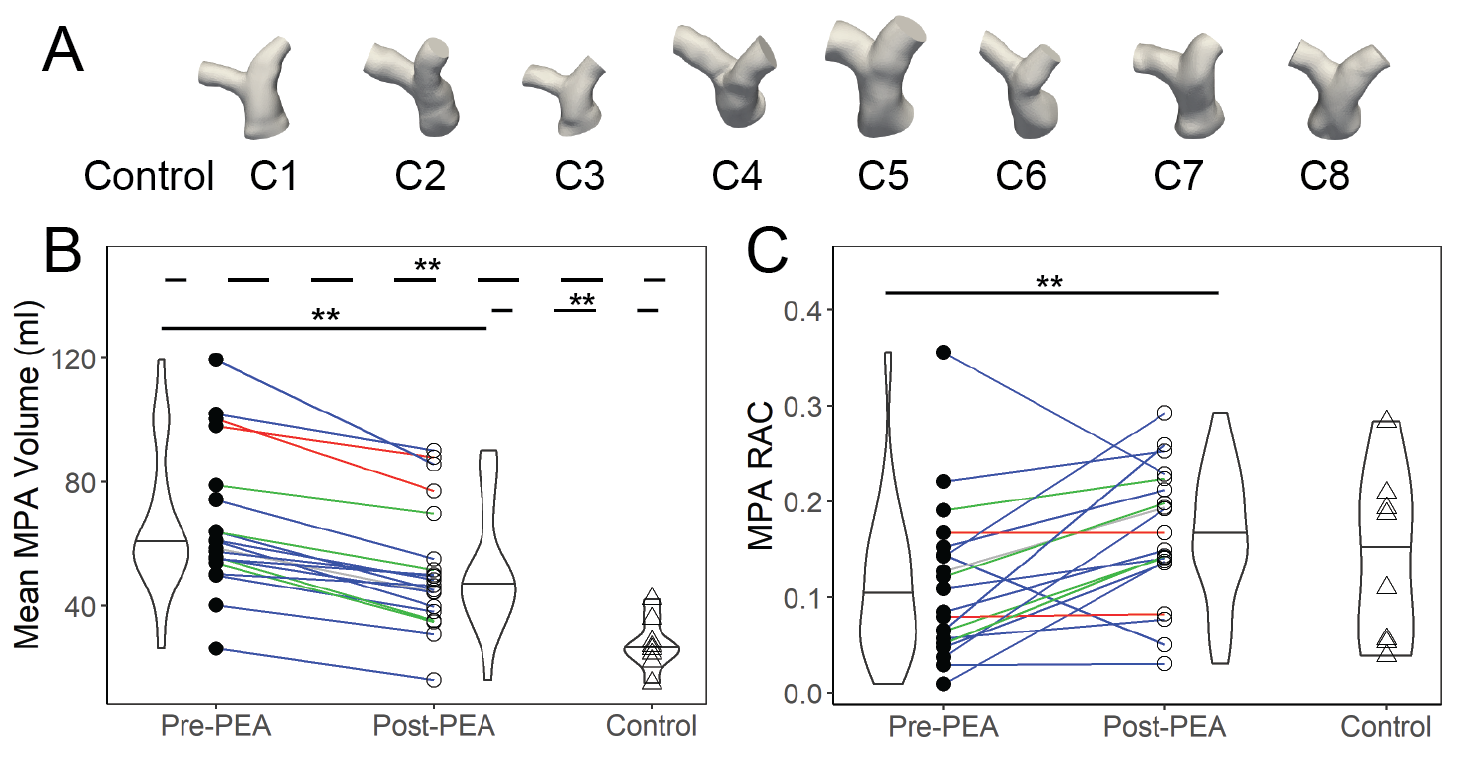


**Additional file 15:** (a) Geometries, (b) volumes, and (c) relative area change in the MPA of patients with CTEPH and the cohort of patients with normal LV/RV and PA function, but with mild to moderate vavulopathies.
