# Supplementary material for: 4D flow cardiovascular magnetic resonance recovery profiles following pulmonary endarterectomy in chronic thromboembolic pulmonary hypertension
Source: J Cardiovasc Magn Reson. 2022 Nov 14;24:59. doi: 10.1186/s12968-022-00893-x (PMC9661778; doi:10.1186/s12968-022-00893-x)
Supplement: Supplementary file 16 — Supplementary Material 16 [file 12968_2022_893_MOESM16_ESM.docx]

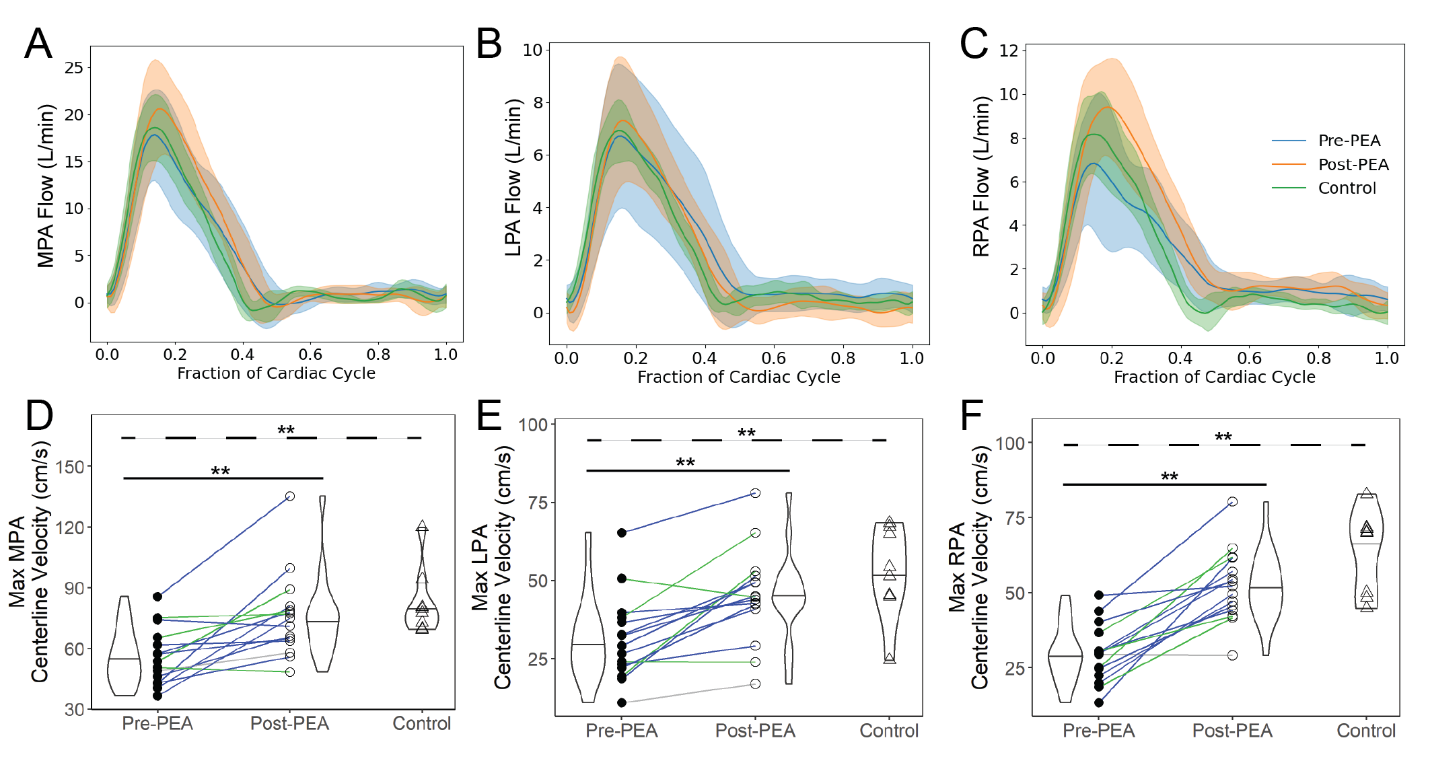


**Additional file 16:** Flow profiles (a- MPA, b- LPA, c- RPA) and velocities (d-MPA, e-LPA, f-RPA) of patients with CTEPH and a cohort of patients with normal LV/RV and PA function, but with mild to moderate vavulopathies.
