# Supplementary material for: 4D flow cardiovascular magnetic resonance recovery profiles following pulmonary endarterectomy in chronic thromboembolic pulmonary hypertension
Source: J Cardiovasc Magn Reson. 2022 Nov 14;24:59. doi: 10.1186/s12968-022-00893-x (PMC9661778; doi:10.1186/s12968-022-00893-x)
Supplement: Supplementary file 17 — Supplementary Material 17 [file 12968_2022_893_MOESM17_ESM.docx]

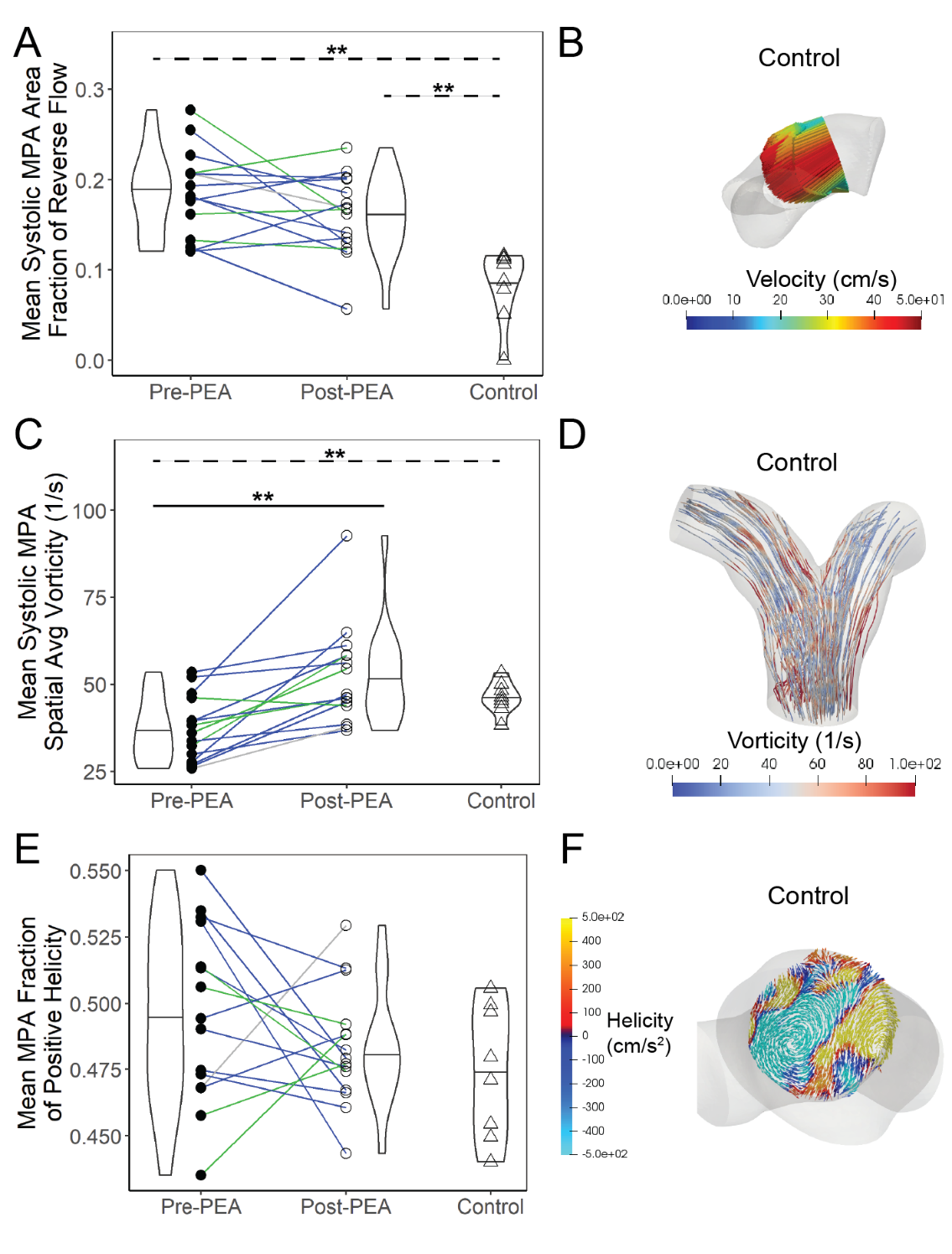


**Additional file 17:** (a) The mean systolic area fraction of reverse flow decreased slightly post-PEA trending towards values in control patients with normal LV/RV and PA function, but with mild to moderate vavulopathies. (b) Visualization of flow direction in a cross-sectional MPA slice in a representative control patient (C3) shows only forward flow. (c) Mean systolic spatially averaged vorticity in the MPA increased post-PEA towards values in control patients with (d) streamlines in a control patient (C4) colored by vorticity showing straightforward velocities with higher vorticity near the walls. (e) The mean fraction of positive helicity was approximately half of the MPA volume for the pre/post-PEA and control group. (f) A cross-section of the MPA shows surface vectors of velocity colored by helicity in a control patient (C5) with distinct rotating clockwise and counterclockwise structures.
